# Superior performance of biofilm versus planktonic *Limosilactobacillus reuteri* in protection of the intestines and brain in a piglet model of necrotizing enterocolitis

**DOI:** 10.1038/s41598-023-44676-5

**Published:** 2023-10-23

**Authors:** Samantha J. Wala, Nitin Sajankila, Mecklin V. Ragan, Audrey F. Duff, Joseph Wickham, Samuel G. Volpe, Yijie Wang, Miriam Conces, Zachary Dumbauld, Nanditha Purayil, Siddharth Narayanan, Adrian Rajab, Belgacem Mihi, Michael T. Bailey, Steven D. Goodman, Gail E. Besner

**Affiliations:** 1 Center for Perinatal Research, Nationwide Children’s Hospital, Columbus, OH USA; 2 Department of Pediatric Surgery, Nationwide Children’s Hospital, 700 Children’s Drive, Columbus, OH 43205 USA; 3 Center for Microbial Pathogenesis, Nationwide Children’s Hospital, Columbus, OH USA; 4 Department of Pathology, Nationwide Children’s Hospital, Columbus, OH USA

**Keywords:** Biofilms, Infant necrotizing enterocolitis, Diseases

## Abstract

Necrotizing enterocolitis (NEC) is the leading cause of gastrointestinal-related death in premature infants. Its etiology is multifactorial, with intestinal dysbiosis playing a major role. Probiotics are a logical preventative therapy for NEC, however their benefits have been inconsistent. We previously developed a novel probiotic delivery system in which planktonic (free-living) *Limosilactobacillus reuteri* (*Lr*) is incubated with biocompatible dextranomer microspheres (DM) loaded with maltose (*Lr*-DM-maltose) to induce biofilm formation. Here we have investigated the effects of *Lr*-DM-maltose in an enteral feed-only piglet model of NEC. We found a significant decrease in the incidence of Definitive NEC (D-NEC), death associated with D-NEC, and activated microglia in the brains of piglets treated with *Lr*-DM-maltose compared to non-treated piglets. Microbiome analyses using 16S rRNA sequencing of colonic contents revealed a significantly different microbial community composition between piglets treated with *Lr*-DM-maltose compared to non-treated piglets, with an increase in Lactobacillaceae and a decrease in Clostridiaceae in *Lr*-DM-maltose-treated piglets. Furthermore, there was a significant decrease in the incidence of D-NEC between piglets treated with *Lr*-DM-maltose compared to planktonic *Lr*. These findings validate our previous results in rodents, and support future clinical trials of *Lr* in its biofilm state for the prevention of NEC in premature neonates.

## Introduction

Necrotizing enterocolitis (NEC) is a devastating intestinal disease that mainly affects premature babies fed preterm formulas. Despite decades of research, minimal improvements have been made in the diagnosis, management, or outcomes of the disease, with mortality remaining as high as 42%^1–3^. Although the etiology of NEC is multifactorial, recent studies have highlighted the significance of intestinal dysbiosis in the pathogenesis of NEC^4^. In contrast to their healthy counterparts, preterm neonates that develop NEC have decreased overall diversity of the intestinal microbiome accompanied by decreased abundance of beneficial anaerobic bacteria such as *Bifidobacterium* and *Bacteroides*, and an increased abundance of *Gammaproteobacteria* and *Clostridium*^5–8^. In the absence of a known cure, increased attention has been placed on the development of prophylactic strategies, including the use of enteral probiotics^9^. Probiotics are defined as “live microorganisms that, when administered in adequate amounts, confer a health benefit on the host”^10^. Given the dysbiosis that exists in patients with NEC, it is possible that enteral administration of probiotics may be able to prevent the disease by increasing healthy gut microbes and improving intestinal microbial diversity^11,12^.

*Limosilactobacillus reuteri* (*Lr*), formerly known as *Lactobacillus reuteri*, is a commensal gut microbe that possesses anti-microbial properties by inducing oxidative stress through the production of reuterin^13^. Futhermore, *Lr* has been shown to possess anti-inflammatory effects via the production of histamine^14^. *Lr* is present in the healthy gut microbiome of term, breastfed neonates^15^, whereas lactic acid-fermenting bacteria are reduced in the microbiome of premature neonates^16^. Interestingly, *Lr* was commonly detected in the human gut in the 1950’s–60’s^17^, but it is less abundant in humans today, leading to an increased predisposition for dysbiosis and gut disorders^18–20^.

We have developed a novel strategy for enteral probiotic administration that capitalizes on the ability of *Lr* to produce a biofilm^21,22^. When *Lr* is grown on the surface of biocompatible dextronamer microspheres (DM), biofilm formation is induced. Furthermore, DMs can be loaded with beneficial substrates such as maltose (*Lr*-DM-maltose), which induces increased biofilm formation^22^. Biofilms protect bacteria against challenging environmental factors including acidic gastric pH, host immune activity, and direct competition with other intestinal microbes^23^. We have previously shown that *Lr*-DM-maltose has significantly improved survival at low pH and increased adherence to intestinal epithelial cells in vitro compared to planktonic *Lr*^22^. We have also demonstrated that *Lr* in its biofilm state has increased efficacy compared to *Lr* in its planktonic (free-living) state in a rat model of NEC^24^.

Survivors of NEC have significant life-long morbidities, including neurodevelopmental delays. Approximately 45% of survivors have some neurodevelopmental sequelae of the disease^25,26^. Importantly, our previous studies showed that administration of *Lr* in its biofilm state prevented neurodevelopmental impairments in rats that survived NEC, with improved spatial learning and memory, and decreased activated microglia and increased myelination in the brain^27^.

These findings taken together demonstrate that *Lr* in its biofilm state can decrease the morbidity and mortality associated with experimental NEC in a rodent model of the disease. Towards our goal of administering *Lr* in its biofilm state to premature babies to prevent NEC, demonstration of efficacy in a large animal model is highly desirable. We have recently developed an enteral feed-only piglet model of NEC^28^. Compared to rat pups, piglets have similar average birthweight to premature human neonates^29^, making them a more ideal model for drug development for this patient population. As opposed to rodent NEC models which require exposure to repeated episodes of stresses such as hypoxia, hypothermia, and addition of lipopolysaccharide (LPS) or pathogenic bacteria added to hypercaloric feeds, piglets develop NEC spontaneously following formula feeding, similar to premature human neonates^30–32^. Piglets also demonstrate characteristic clinical signs and histopathologic features observed in human neonates with NEC, thereby replicating hallmarks of this disease^33^. In addition, they can develop pneumatosis within the bowel wall that can be observed radiographically^29,33–35^. These premature animals can also receive similar interventions that are performed on human neonates in the NICU^33^. Furthermore, pigs also share some features of the human microbiome, making these animals a strong model for digestive disease^36,37^. We previously demonstrated an abundance of Gammaproteobacteria in piglets exposed to NEC-inducing conditions^28^. Gammaproteobacteria are similarly increased in preterm infants with NEC^38^.

Using our enteral feed-only model of NEC in piglets^28^, we now demonstrate that enteral administration of *Lr* in its biofilm state has a positive impact on the intestine, the microbiome, and the developing brain. In addition, *Lr* in its biofilm state has a greater effect against NEC compared to *Lr* in its planktonic state.

## Results

### *Lr* in its biofilm state protects the intestines from NEC

We first evaluated the benefits of administering *Lr*-DM-maltose (*Lr* in its biofilm state) on experimental NEC in piglets. Only one piglet in this experiment died within the first 24 h after birth, and was in the Colostrum group. Piglets that survived the first 24 h of life were randomized to the following groups: Group 1: Colostrum only (negative control; N = 20), Group 2: NEC + Saline (positive control; N = 45), and Group 3: NEC + *Lr*-DM-maltose (treatment; N = 47). The average birth weights for the Colostrum, NEC + Saline, and NEC + *Lr*-DM-maltose groups were 0.902 kg (0.304–1.350 kg), 0.906 kg (0.415–1.489 kg), and 0.969 kg (0.495–1.449 kg), respectively. The average percent weight change in each experimental group over the course of the experiment is shown in Supplementary Fig. S1.

At least 3 of the following 4 criteria were required for a piglet to be diagnosed with definitive NEC (D-NEC): clinical sickness score (CSS) of ≥ 5 out of 8 in the last 12 h of life, gross injury score of ≥ 4 out of 6, histologic injury score of ≥ 3 out of 5, and bacterial translocation to ≥ 2 internal organs (liver, spleen, and mesenteric lymph nodes with ≥ 1 colony-forming unit (CFU)/mg of tissue)^28^.

#### Clinical sickness scores

**Figure 1:**
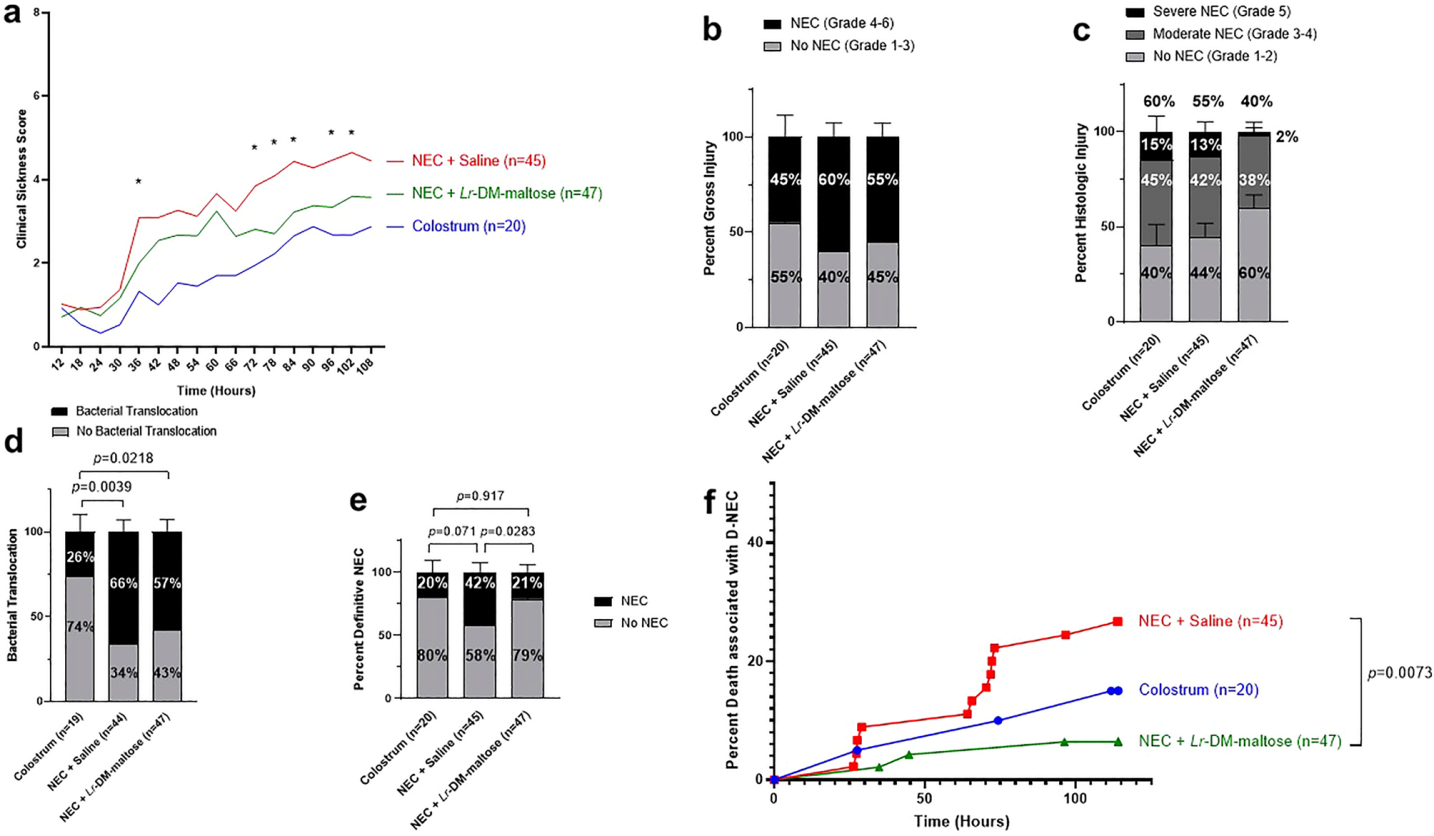
Effect of *Lr*-DM-maltose in a piglet model of necrotizing enterocolitis (NEC). In Experiment 1, piglets were randomized to Colostrum (N = 20), NEC + Saline (N = 45), and NEC + *Lr*-DM-maltose (N = 47) groups. The following data represent the combined results of 7 independent experiments. (**a**) Clinical Sickness Scores (CSS), *p < 0.05, (**b**) gross injury scores, **(c)** histologic injury scores, (**d**) bacterial translocation, (**e**) Definitive NEC (D-NEC) score, and (**f**) percent death associated with D-NEC. CSS was evaluated using 2-way analysis of variance (ANOVA). Columns indicate group means and error bars show standard error of the mean (SEM). The Kruskal–Wallis test was performed for gross injury scores, histologic injury scores, bacterial translocation, and D-NEC scores. Percent death associated with D-NEC was analyzed using the log-rank (Mantel-Cox) test with Bonferroni correction.

The Colostrum group had the lowest CSS throughout the experiment, whereas the NEC + Saline group had the highest CSS. The NEC + *Lr*-DM-maltose group had significantly decreased CSS compared to the NEC + Saline group at the 36, 72, 78, 84, 96, and 102 h experimental time points (*p* < 0.05) (Fig. 1a).

#### Gross injury scores

**Figure 2:**
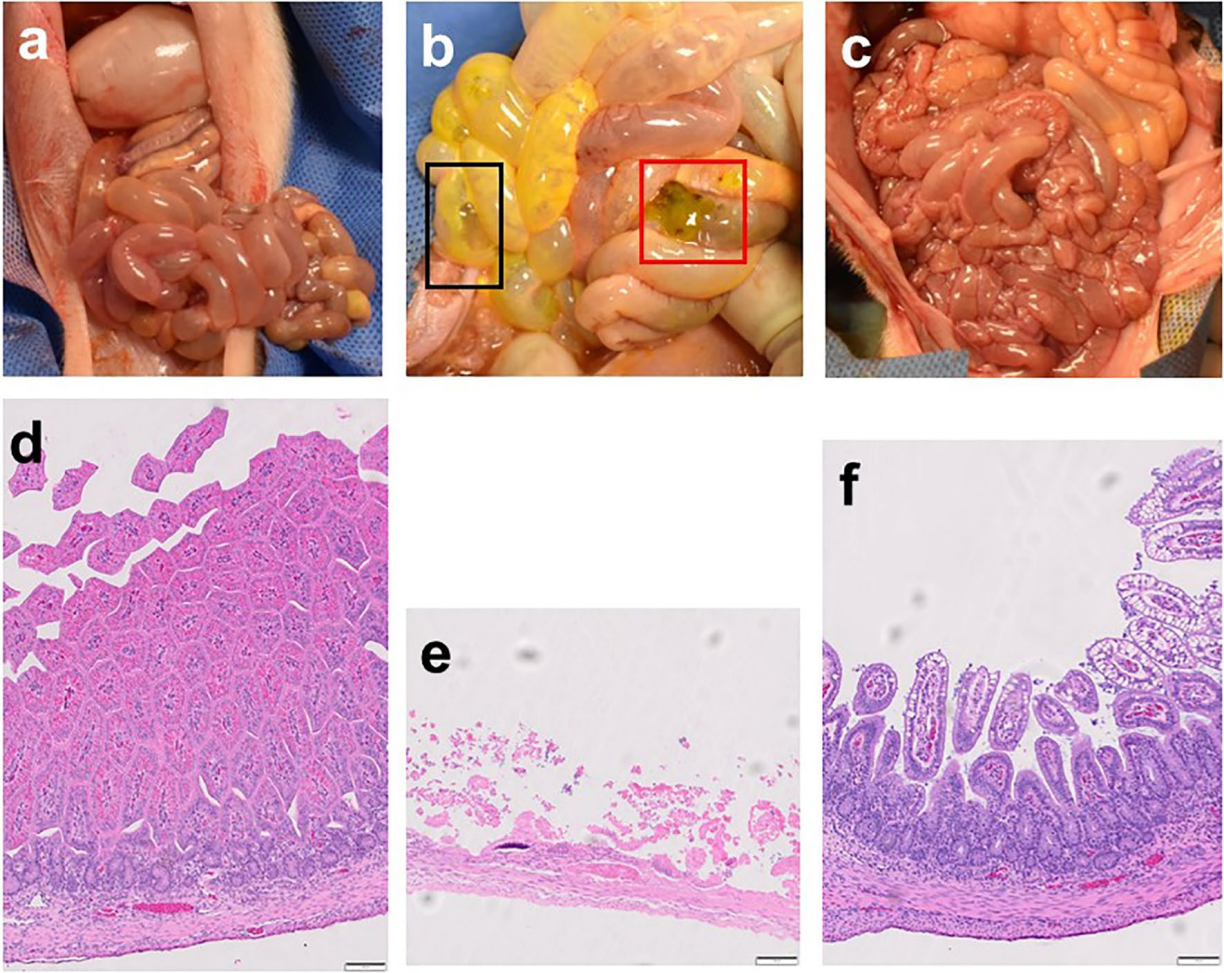
Gross and histologic images of intestine from Experiment 1. Shown are gross images of small bowel and colon from piglets in the (**a**) Colostrum, (**b**) NEC + Saline, and (**c**) NEC + *Lr*-DM-maltose groups. In panel (**b**), the black inset highlights pneumatosis in the small bowel wall and the red inset demonstrates colonic perforation. Also shown are histologic images of small bowel stained with hematoxylin and eosin (H&E) at 10× magnification from piglets in the (**d**) Colostrum, (**e**) NEC + Saline, and (**f**) NEC + *Lr*-DM-maltose groups.

At necropsy, the Colostrum group had the lowest gross injury consistent with NEC (45%) compared to the NEC + Saline group (60%) and the NEC + *Lr*-DM-maltose groups (55%) (Fig. 1b). There were no statistically significant differences between any of these groups. Gross appearances of small bowel and colon in the Colostrum, NEC + Saline, and NEC + *Lr*-DM-maltose groups, are shown in Fig. 2a–c. In the NEC + Saline piglet, there were severe lesions of the intestine, including pneumatosis in the wall of the small bowel and colonic perforation. This contrasts with the healthy-appearing bowel in the Colostrum and NEC + *Lr*-DM-maltose groups.

#### Histologic injury scores

The least amount of histologic injury consistent with NEC was observed in the NEC + *Lr*-DM-maltose group (40%) compared to the Colostrum group (60%) and the NEC + Saline group (55%) (Fig. 1c). However, these differences were not statistically significant. Histologic images of small bowel are shown in Fig. 2d–f, demonstrating transmural necrosis in the NEC + Saline group, and intact villi in the Colostrum and NEC + *Lr*-DM-maltose groups.

#### Bacterial translocation

There was significantly increased bacterial translocation in the NEC + Saline group and the NEC + *Lr*-DM-maltose group compared to the Colostrum group (66% vs. 26%, *p* = 0.0039 and 57% vs. 26%, *p* = 0.0218, respectively) (Fig. 1d). Although there was a reduction in bacterial translocation in the NEC + *Lr*-DM-maltose group compared to the NEC + Saline group, this was not statistically significant (57% vs. 66%).

#### Definitive NEC (D-NEC)

The incidence of D-NEC in the Colostrum group was 20%. This increased significantly to 42% in the NEC + Saline group (42% vs. 20%, *p* = 0.0283) (Fig. 1e). The incidence of D-NEC in the NEC + *Lr*-DM-maltose group was half of that in the NEC + Saline group (21% vs. 42%, *p* = 0.0283). The incidence of D-NEC in the *Lr*-DM-maltose group was comparable to that of the healthy Colostrum group (21% vs. 20%, respectively).

#### Death associated with D-NEC

Death associated with D-NEC after controlling for death unrelated to NEC was determined. The highest percent of pigs that died in association with D-NEC was found in the NEC + Saline group (Fig. 1f). Compared to the NEC + Saline group, death associated with D-NEC was decreased in the Colostrum group, and further decreased significantly in the NEC + *Lr*-DM-maltose group (NEC + Saline vs. NEC + *Lr*-DM-maltose, *p* = 0.0073). There was no signficiant difference in terms of death associated with D-NEC in the Colostrum group compared to the NEC + Saline group, or in the Colostrum group compared to the NEC + *Lr*-DM-maltose group.

### *Lr* in its biofilm state decreases inflammation in the prefrontal cortex (PFC) during NEC

**Figure 3:**
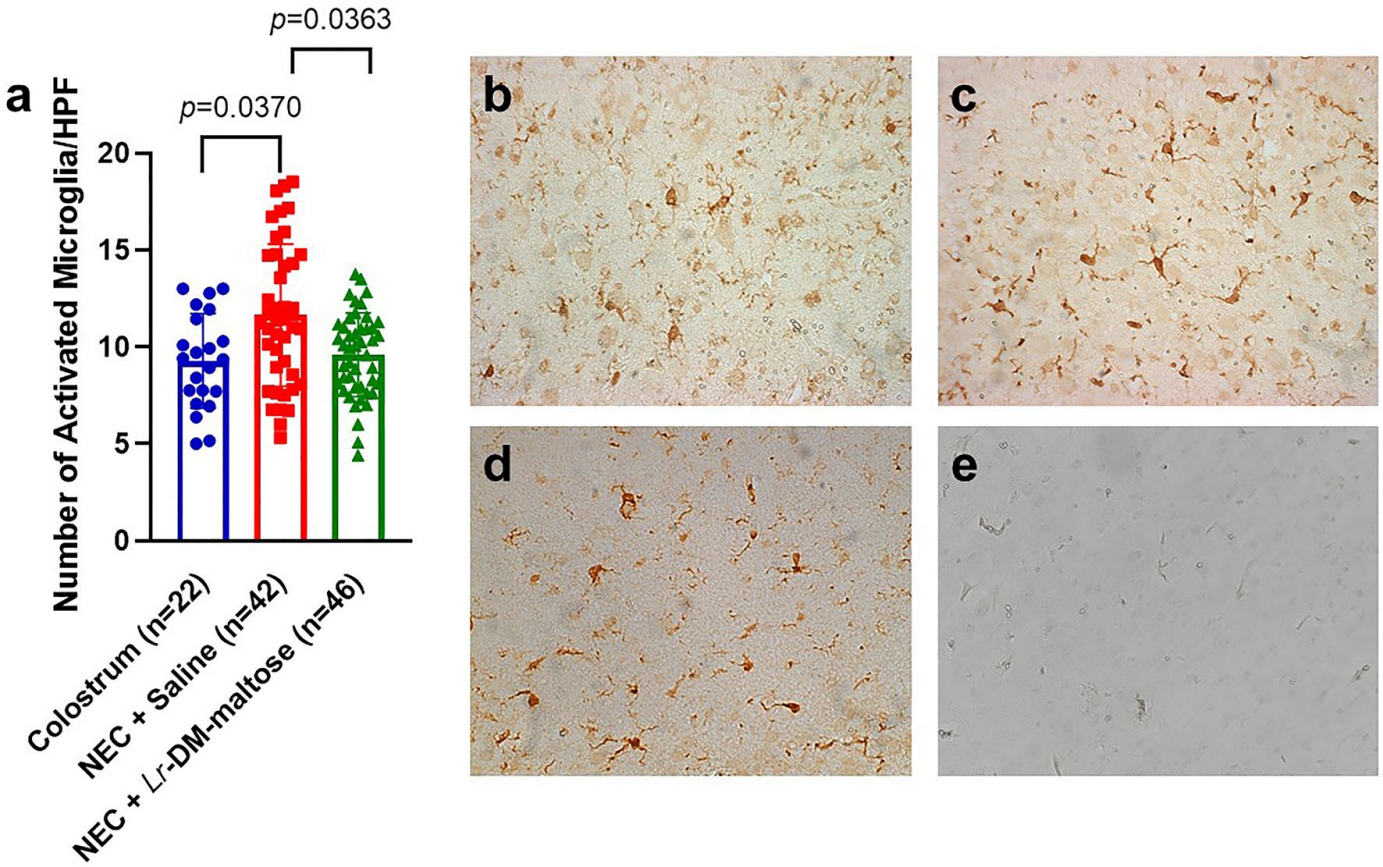
Activated microglia in the prefrontal cortex (PFC) from Experiment 1. (**a**) Mean count of activated microglia in PFC. Points indicate counts for each individual animal, columns indicate the mean, and error bars show standard error of the mean (SEM). The Kruskal–Wallis test was performed. Representative images of Iba-1 stained activated microglia at 40× magnification in the (**b**) Colostrum, (**c**) NEC + Saline, and (**d**) NEC + *Lr*-DM-maltose groups. (**e**) represents the negative control.

Anti-ionized calcium binding adaptor molecule 1 (Iba-1) is a known marker for activated microglia^39^. Piglets in the NEC + Saline group had significantly increased average number of activated microglia in the prefrontal cortex (PFC) compared to the Colostrum group (11.64 vs. 9.28, *p* = 0.0370) (Fig. 3). Treatment with *Lr*-DM-maltose significantly decreased the average number of activated microglia compared to the NEC + Saline group (9.60 vs. 11.64, *p* = 0.0363).

Myelination within the PFC was also investigated. As shown in Supplementary Fig. S2a, there was no significant difference in myelin basic protein (MBP) expression in the PFC between the Colostrum, NEC + Saline, and NEC + *Lr*-DM-maltose groups.

### *Lr* in its biofilm state alters beta diversity but not alpha diversity

**Figure 4:**
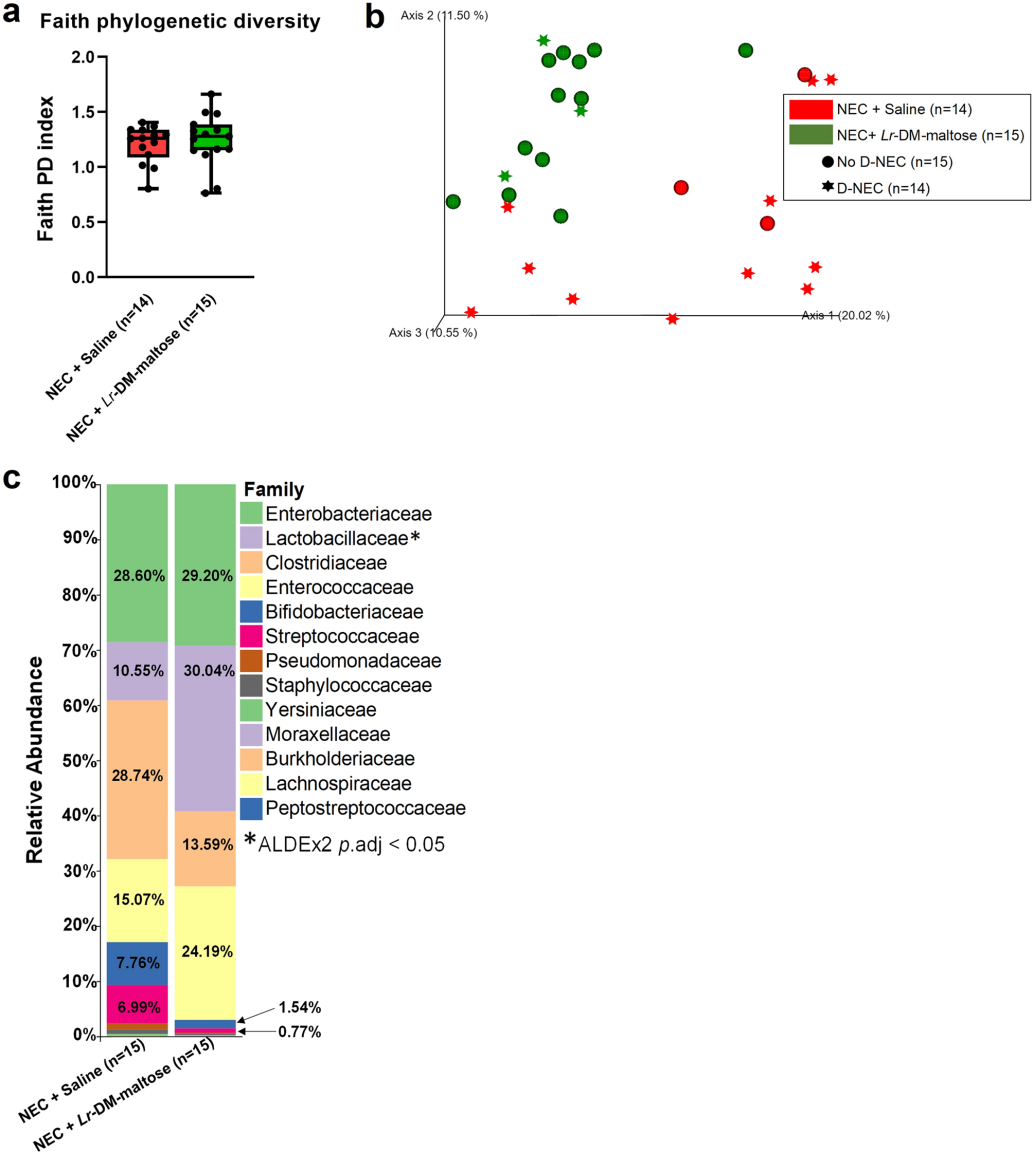
Microbiome analyses. (**a**) Faith phylogenetic diversity within treatment (*p* = 0.663). Pairwise comparisons were analyzed by the Kruskal–Wallis test (*p* < 0.05) in QIIME 2.0. Box and whisker plots denote minimum, maximum, and interquartile range. (**b**) Jaccard distance beta diversity within treatment (*p* = 0.001) and occurrence of Definitive NEC (D-NEC) (*p* = 0.063). Beta diversity was analyzed by PERMANOVA with 999 randomizations of the data in QIIME 2.0. (**c**) Family level relative abundance within treatment. Differences were assessed with ALDEx2 in R, and Wilcoxon *p* values were Benjamini–Hochberg adjusted (*p*.adj = 0.05).

The effects of probiotic administration on piglet microbiome alpha diversity were measured with Faith phylogenetic diversity (Faith PD), however, no differences were detected between NEC + Saline and NEC + *Lr*-DM-maltose when assessed by Faith PD (Fig. 4a) or by supplemental alpha diversity metrics (see Supplementary Fig. S3 online). In contrast, probiotic administration did significantly alter beta diversity which was visualized by plotting the Jaccard distance matrix (Fig. 4b). As denoted by red or green shapes, similar clustering patterns were noted within but not between treatments, and indicated distinct community compositions between piglets in the NEC + Saline versus the NEC + *Lr*-DM-maltose groups (PERMANOVA, 999 permutations; *p* = 0.001). Similar statistical findings were also noted for supplementary beta diversity metrics (see Supplementary Fig. S4 online). While no significant differences were detected between No D-NEC (spheres) and D-NEC piglets (stars), there was a trend towards significance (PERMANOVA, 999 permutations; *p* = 0.063) and a pattern wherein piglets with D-NEC in the NEC + *Lr*-DM-maltose group clustered away from piglets with D-NEC in the NEC + Saline group (Fig. 4b). Thus, probiotic administration significantly influenced microbial composition relative to Saline-treated piglets, and appeared to limit NEC-associated shifts in community composition between D-NEC and No D-NEC piglets, which were more pronounced in the NEC + Saline group.

### Probiotic administration is associated with a modification of microbiome composition

Treatment-specific relative abundance values were determined at the family level (Fig. 4c). Relative abundance bar plots for individual piglets categorized by treatment or occurrence of NEC are available in Supplementary Fig. S5–S9. Significant differences in relative abundance were assessed using ALDEx2 with Wilcoxon *p*.adj. < 0.05 to denote significance (see Supplementary File online). Here, Lactobacillaceae was the only statistically significantly different taxa (*p.*adj = 0.008; Fig. 4c) and was more abundant in NEC + *Lr*-DM-maltose (30.04%) relative to NEC + Saline (10.55%). Total *L. reuteri* was significantly higher in the NEC + *Lr*-DM-maltose group compared to the NEC + Saline group, and total bacteria between both groups was unchanged (see Supplementary Fig. S10 online). Additionally, there was an approximate 10% increase (*p.*adj > 0.05) in Enterococcaceae in NEC + *Lr*-DM-maltose relative to Saline controls (24.19% versus 15.07%, respectively). In contrast, Clostridiaceae abundance was more than twice as high (*p.*adj > 0.05) in NEC + Saline pigs (28.74%) than in NEC + *Lr*-DM-maltose (13.59%) piglets. Overall, taxonomic findings are in line with the fact that the probiotic consists of *Lr*, and further demonstrate that daily oral administration of the *Lr*-DM-maltose probiotic resulted in a more lasting and measurable increase in resident *Lactobacillus*.

### *Lr* in its biofilm state is superior to planktonic *Lr* in protection of the intestines from NEC

We next evaluated the efficacy of *Lr* in its biofilm state relative to *Lr* in its planktonic state on protection of the intestines from NEC. Only 1 piglet in this experiment died within the first 24 h after birth, and was in the NEC + *Lr*-DM-maltose group. Piglets that survived the first 24 h of life were randomized to the following groups: Group 1) NEC + Saline (positive control; N = 15), Group 2) NEC + *Lr* (planktonic) (N = 16), and Group 3) NEC + *Lr*-DM-maltose (treatment; N = 15). The average birth weights for the NEC + Saline, NEC + *Lr* (planktonic), and NEC + *Lr*-DM-maltose groups were 1.075 kg (0.588–1.464 kg), 1.068 kg (0.715–1.405 kg), and 1.046 kg (0.695–1.274 kg), respectively. The average percent weight change in each experimental group over the course of the experiment is demonstrated in Supplementary Fig. S11.

#### Clinical sickness scores

**Figure 5:**
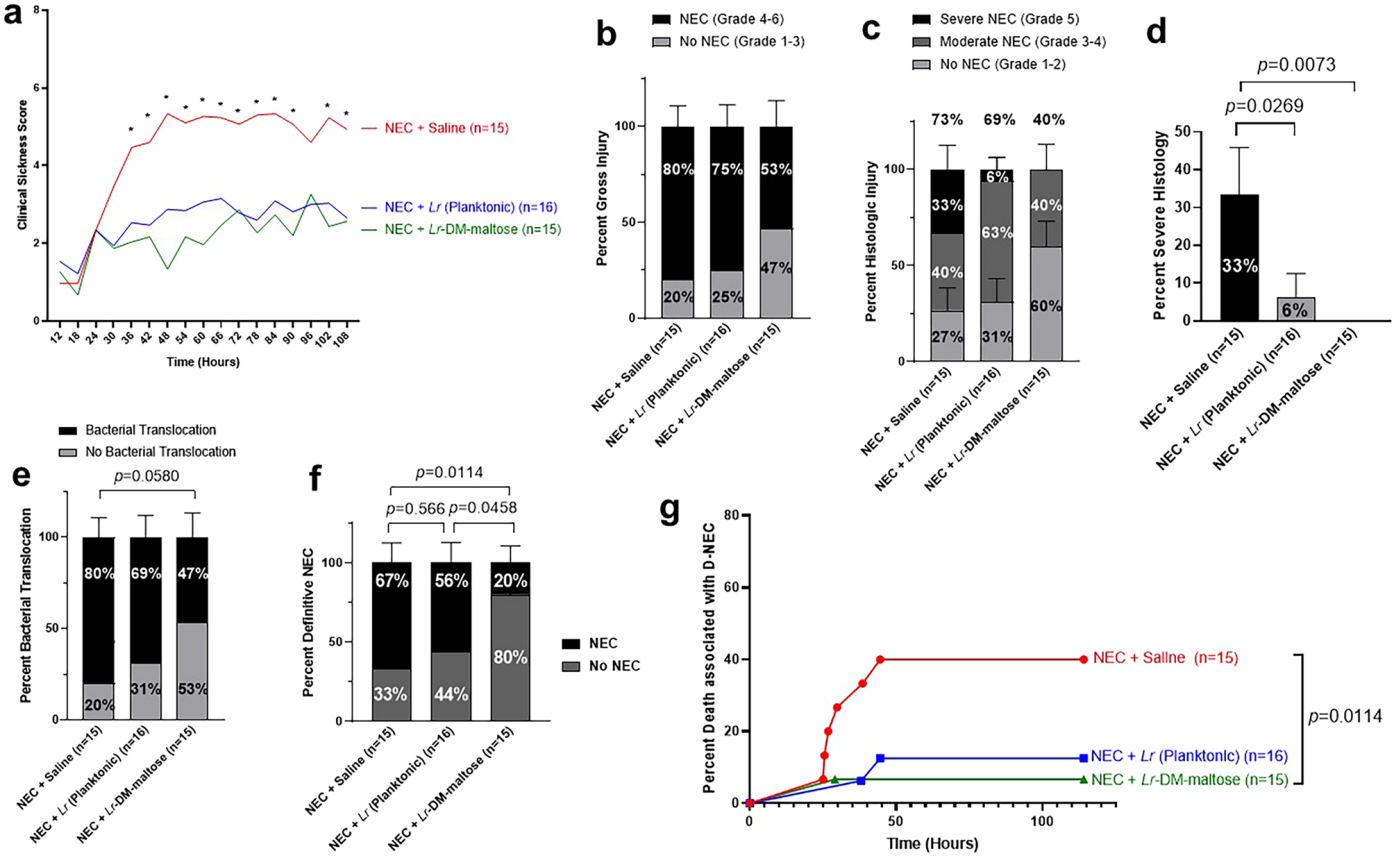
Comparison of *Lr* (planktonic) and *Lr*-DM-maltose in a piglet model of necrotizing enterocolitis (NEC). In Experiment 2, piglets were randomized to NEC + Saline (N = 15), NEC + *Lr* (planktonic) (N = 16), NEC + *Lr*-DM-maltose (N = 15) groups. The following data represent the combined results of 3 independent experiments. (**a**) Clinical Sickness Scores (CSS), *p < 0.05, (**b**) gross injury scores, (**c**) histologic injury scores, (**d**) severe histologic injury, (**e**) bacterial translocation, (**f**) Definitive NEC (D-NEC) score, and (**g**) percent death associated with D-NEC. Statistical significance was calculated using 2-way analysis of variance (ANOVA) for CSS. Columns indicate group means and error bars show standard error of the mean (SEM). The Kruskal–Wallis test was performed for gross injury scores, histologic injury scores, severe histologic injury, bacterial translocation, and D-NEC score. Percent death associated with D-NEC was analyzed using the log-rank (Mantel-Cox) test with Bonferroni correction.

The NEC + Saline group had significantly higher CSS at 36, 42, 48, 54, 60, 66, 72, 78, 84, 90, 102, and 108 h compared to both the NEC + *Lr* (planktonic) and NEC + *Lr*-DM-maltose groups (Fig. 5a). Piglets in the NEC + *Lr* (planktonic) and NEC + *Lr*-DM-maltose groups had similar CSS throughout the experiment.

#### Gross injury scores

**Figure 6:**
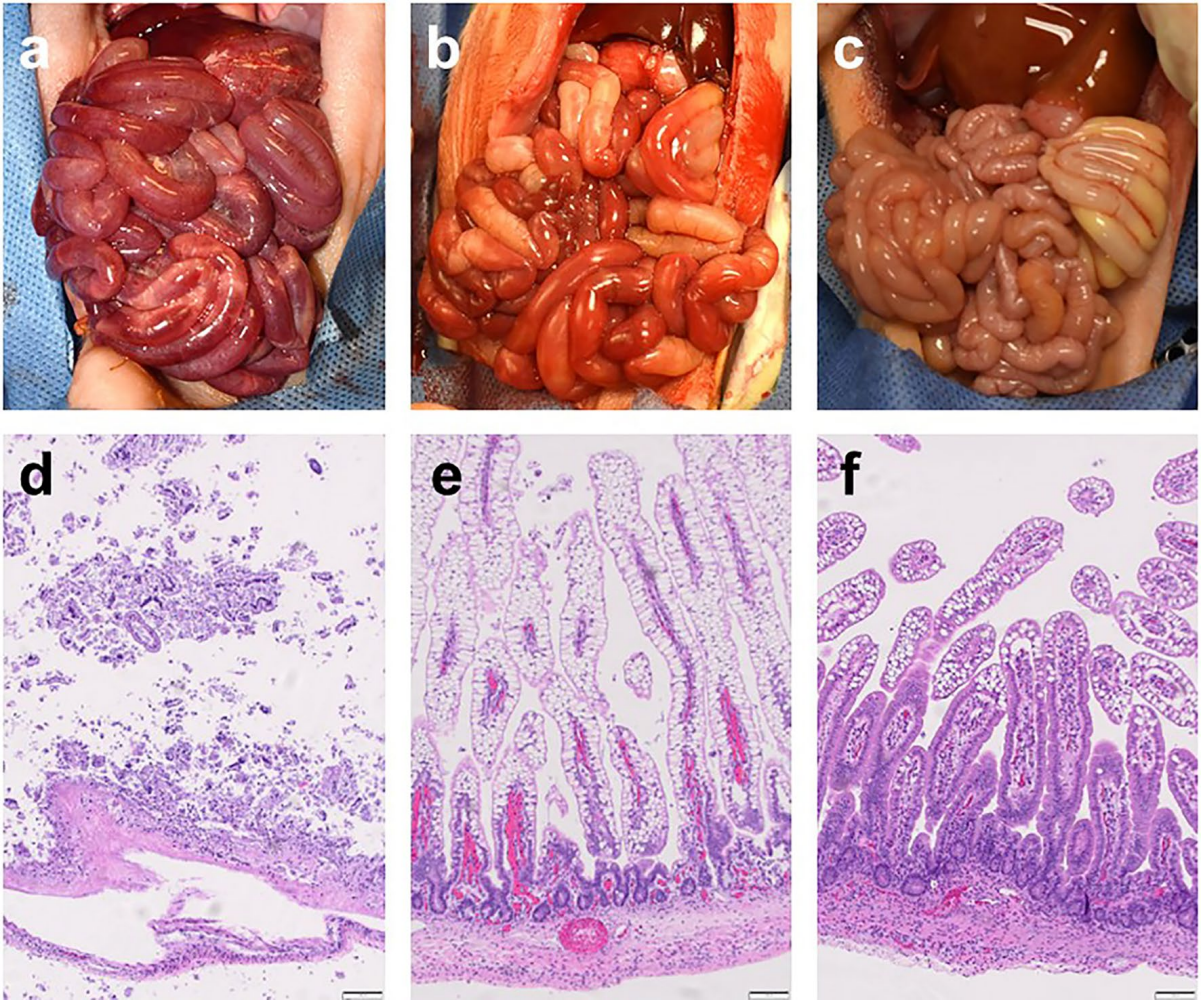
Gross and histologic images of intestine from Experiment 2. Shown are gross images of small bowel and colon from piglets in the (**a**) NEC + Saline, (**b**) NEC + *Lr* (planktonic), and (**c**) NEC + *Lr*-DM-maltose groups. Also shown are histologic images of small bowel intestinal epithelium stained with hematoxylin and eosin (H&E) at 10× magnification from piglets in (**d**) NEC + Saline, (**e**) NEC + *Lr* (planktonic), and (**f**) NEC + *Lr*-DM-maltose groups.

The NEC + *Lr*-DM-maltose group had the lowest incidence of gross injury consistent with NEC (53%) compared to both NEC + Saline (80%) and NEC + *Lr* (Planktonic) (75%) (Fig. 5b). This difference was not statistically significant. Differences in gross injury between representative animals from each group are shown in Fig. 6a–c. There is extensive hemorrhage throughout the small bowel in the images from the NEC + Saline and NEC + *Lr* (planktonic) groups, whereas the small bowel and colon appear healthy in the image from the NEC + *Lr*-DM-maltose piglet.

#### Histologic injury scores

The NEC + Saline group had the highest incidence of histologic injury consistent with NEC (73%) (Fig. 5c). This was decreased slightly in the NEC + *Lr* (planktonic) group (69%), and was further decreased in the NEC + *Lr*-DM-maltose group (73% vs. 40%, *p* = 0.0643). Significant differences were identified in severe histologic injury (Grade 5 injury). The NEC + Saline group had the highest incidence of severe histologic injury (33%) (Fig. 5d). This decreased to 8% in the NEC + *Lr* (planktonic) group (*p* = 0.0269), and was eliminated (0%) in the NEC + *Lr*-DM-maltose group (*p* = 0.0073). Histologic images of small bowel from each of the groups are shown in Fig. 6d–f. The piglet in the NEC + Saline group displayed patchy villous necrosis and pneumatosis while the piglet treated with *Lr* (planktonic) had blunted villi. On the other hand, the villi and crypts were mostly intact in the piglet treated with *Lr*-DM-maltose.

#### Bacterial translocation

Piglets in the NEC + Saline group had the highest incidence of bacterial translocation (80%) (Fig. 5e). Bacterial translocation decreased slightly to 69% in the NEC + *Lr* (planktonic) group, and decreased further to 47% in the NEC + *Lr*-DM-maltose group (80% vs. 47%).

#### Definitive NEC (D-NEC)

Piglets in the NEC + Saline group had the highest incidence of D-NEC (67%) (Fig. 5f). D-NEC was slightly decreased to 56% in the NEC + *Lr* (planktonic) group, and significantly decreased to 20% in the NEC + *Lr*-DM-maltose group (*p* = 0.0114). There was a significant difference in D-NEC between the NEC + *Lr* (planktonic) group and the NEC + *Lr*-DM-maltose group (56% *vs.* 20%, *p* = 0.0458).

#### Death associated with D-NEC

The highest percent of pigs that died in association with D-NEC was found in the NEC + Saline group (Fig. 5g). There was a decrease in death associated with D-NEC in the NEC + *Lr* (planktonic) group (not statistically significant). There was a further, statistically significant, decrease in the NEC + *Lr*-DM-maltose group (NEC + Saline *vs.* NEC + *Lr*-DM-maltose, *p* = 0.0114). There was no significant difference in terms of death associated with D-NEC in the NEC + *Lr*-DM-maltose group compared to the NEC + *Lr* (planktonic) group.

## Discussion

There are currently no prophylactic treatments that consistently decrease the incidence of NEC in the vulnerable preterm patient population. In this study, we describe a novel biofilm-based delivery system for *Lr*, which has positive effects in an enteral-feed only piglet model of NEC. This is the first study that describes the benefits of using a biofilm-based probiotic compared to the probiotic in its planktonic form for the prevention of NEC. The study was conducted in preterm piglets fed formula containing maltodextrin, which is known to spontaneously induce NEC.

Using a rat model of NEC, we have previously shown that *Lr* in its biofilm state was associated with a reduction in the incidence and severity of NEC, improvement of NEC-associated dysbiosis, reduced intestinal permeability, and increased survival^40^. In the current study, we now demonstrate that *Lr* in its biofilm state significantly reduces the incidence of D-NEC compared to planktonic *Lr* or to no treatment (Fig. 5f). In piglets, we believe that D-NEC (which includes combined components of clinical sickness score, gross injury, histologic injury, and bacterial translocaton) better represents the complex disease process of NEC, rather than the aforementioned components considered individually. The ability to observe similar benefits in both rodent and porcine animal models of NEC demonstrates the robustness of this therapeutic approach across mammalian species regardless of method of NEC induction, and without negative consequence. In addition, there was a significant decrease in death associated with D-NEC in piglets treated with *Lr* in its biofilm state—an effect that was not observed with planktonic *Lr*. This is an important finding since it demonstrates the superiority of *Lr* in its biofilm state compared to planktonic *Lr* in preventing death associated with NEC. The beneficial effects of *Lr* in its biofilm state are consistent with the known positive effects of biofilm formation. The biofilm state has been associated with increased intestinal bacterial colonization after enteral administration^41,42^. Moreover, *L. reuteri*-derived exopolysaccharide, a component of the biofilm, has immunomodulating properties^43^. Exopolysaccharide in lactobacilli has been shown to decrease pro-inflammatory cytokine and toll-like receptor 4 (TLR4) expression induced by *Salmonella* Typhimurium^43^.

Breakdown of the gut barrier has been associated with a predisposition for NEC in premature infants^44^. In the current study, we observed a trend towards decreased bacterial translocation in the NEC + *Lr*-DM-maltose group compared to the NEC + Saline group. Other investigators have demonstrated decreased bacterial translocation in different animal models using probiotics. In a rat model of short bowel syndrome, daily administration of *Bifidobacterium lactis* reduced the risk of bacterial translocation by 43%^45^. Another group challenged preterm rabbit pups with pathogenic *Enterobacter cloacae* (*E. cloacae*)^46^. Animals that received feeds supplemented with *Lactococcus lactis* subspecies *lactis* had significantly less *E*. *cloacae* translocation to the liver compared to control animals. Lactic acid-producing species have been shown to positively modulate the intestinal epithelial barrier by inducing tight junction proteins in vitro and in *vivo*^47,48^. A recent study from Lee et al.^49^ showed that the strain *Lr* DS0384 accelerates small intestinal and colon maturation in infant mice, which is important for structural integrity.

While the multifactorial etiology of NEC remains incompletely defined, an increasing body of evidence indicates a significant contribution of the neonatal gut microbiome^8,50–52^. Numerous perinatal factors are known to influence development of the infant microbiome including gestational age, delivery mode, antibiotic use, and feeding method^53,54^, many of which have been independently identified as risk factors associated with the development of NEC as reviewed by Pammi et al.^52^. In particular, preterm births and prolonged formula feeding have been associated with adverse pioneer colonization characterized by decreased community diversity and expansion of opportunistic pathogens^55–57^, which reportedly contribute to the onset of human neonatal NEC^8,58,59^. Our results recapitulated these trends as demonstrated by the 15.15% higher relative abundance of *Clostridiaceae* (*p.*adj = 0.418), a family containing potential opportunistic pathogens, in piglets that received formula without supplementation of *Lr* in its biofilm state (28.74% in NEC + Saline piglets versus 13.59% in NEC + *Lr*-DM-maltose piglets). Importantly, as denoted by beta diversity, formula supplementation with *Lr* in its biofilm state resulted in distinct microbial compositions relative to saline administration alone, which likely contributed to reduction in the observed incidence of D-NEC. This in line with results from a randomized, double-blind, placebo-controlled, multi-center trial that demonstrated significantly different bacterial community composition (β-diversity) between extremely low birth weight (birthweight < 1,000 g) preterm infants treated with daily *Lr* DSM 17,938 or placebo^12^. The same study showed no significant effect on NEC using planktonic *L. reuteri*, which is in line with our findings (Fig. 5f).

In contrast to piglets treated with *Lr* in its biofilm state, which expectedly possessed significantly higher levels of Lactobacillaceae, piglets receiving saline only harbored over twice as much Clostridiaceae than probiotic-treated counterparts. This finding is consistent with human studies that have reported an increased prevalence of *Clostridium* in formula-fed infants^60–62^ and moreover, recapitulate the work of Liu et al.^63^ who showed that an oral dosing regimen of *Lr* reduced the prevalence of *Clostridium* spp. in formula-fed piglets. Additionally, multiple groups have observed an association between *Clostridium* abundance and risk or severity of neonatal NEC^7,38,64,65^, which is in agreement with our findings of D-NEC incidence in saline-treated piglets. *Clostridium* are commensal intestinal bacteria, however, conditions such as low community diversity, immature gut physiology, and enteral formula feeding may favor over-proliferation and enhanced pathogenesis of this bacteria^66^. A more recent retrospective study correlated the presence of toxin perfringolysin O gene, *pfoA*, found in typical, virulent infant-associated *C*. *perfringens*, including *C. perfringens*-associated NEC, with increased cell toxicity^67^*. Lr* probiotic formulations have been shown to effectively reduce the severity of a variety of gut bacterial infections, including *Clostridium*, via antimicrobial products and reduction of luminal pH^68^. It is therefore possible that our *Lr* probiotic formulation competitively inhibited the over-proliferation of Clostridiaceae in our model, thereby limiting the overall incidence of D-NEC in those piglets.

We also noted an approximately 10% increase in the abundance of Enterococcaceae in piglets treated with *Lr* in its biofilm state. While some groups have observed increased levels of *Enterococcus* in NEC patients^59^, others have found a greater association of this genera in healthy patients^69^ and noted contrasting pathogenic versus protective effects that may be strain-specific^70^. It is therefore possible that our *Lr* probiotic similarly altered the intestinal environment in a manner that promoted establishment of beneficial strains of *Enterococcus* capable of lowering overall D-NEC incidence.

It is known that neonates who survive NEC are at risk for neurodevelopmental impairment, including cognitive and psychomotor impairment^71^. The PFC plays a dynamic role in emotional regulation, memory storage, behavioral flexibility, and attention^72^. We demonstrated a significant decrease in activated microglia in the PFC of piglets treated with *Lr* in its biofilm state compared to Saline-treated piglets. It has previously been shown that intestinal TLR4 can activate microglia in the brain and result in cognitive dysfunction in a mouse model^73^. Therefore, the ability to decrease microglial activation using *Lr* in its biofilm state may have protective cognitive effects. Decreased myelination in the brains of human infants and mice with NEC has also been described^73^. However, we did not observe a change in MBP expression in the PFC. Furthermore, we did not observe a difference in MBP expression between the Colostrum group and the Saline-treated NEC group, despite increased inflammation within the latter group as demonstrated by increased activated microglia. Aberrant myelination has been associated with NEC in other experimental animal models^74^. It may be that the premature age and short lifespan of our piglets after NEC induction did not allow for a difference in MBP expression to be detected. Similarly, another group of investigators did not observe a difference in cerebral myelination in premature piglets with varying degrees of NEC severity^75^.

There are several limitations related to this study. We did not specifically study the mechanism(s) by which *Lr* in its biofilm state prevents NEC in our piglet model. However, our past studies of this probiotic in a rodent model have demonstrated the importance of the production of both reuterin and histamine by *Lr* in preventing NEC^24^. Furthermore, the delayed manifestations and subsequent clinical effects of *Lr* in its biofilm state on neurodevelopment in premature piglets was not assessed in this study. The ability to perform cognitive tests on premature piglets is significantly more challenging from a resources standpoint compared to rodents.

While the rationale for the use of probiotics is clear, the efficacy of enteral probiotics in preventing NEC in human neonates has been variable^76^. In fact, the American Association of Pediatrics has suggested that, at this stage, probiotics should not be routinely used to prevent NEC due to concerns of probiotic-related sepsis, contamination or poor manufacturing, and questionable effects in the preterm neonatal population, particularly those weighing < 1000 g^77^. This has increased the need for more novel strategies for probiotic administration to capitalize on the beneficial effects of these microbes while minimizing the risks of probiotic-related sepsis. In this study, we provide data to support the use of *Lr* in its biofilm state for the prevention of NEC using a piglet model of the disease.

We have produced Good Manufacturing Practice (GMP)-grade *Lr* in its biofilm state (SB-121; Scioto Biosciences, Inc.) designed for human administration. A recent phase 1b clinical trial demonstrated the safety and tolerability of SB-121 in adults with autism spectrum disorder (ASD)^78^. NEC and ASD are both diseases that involve the gut-brain axis^79,80^. Complex interactions between the gut microbiota and the brain can affect neurodevelopment in both diseases. Strikingly, the recent ASD study showed that SB-121 was not only well-tolerated, but that it significantly improved adaptive behavior as measured by Vineland-3 Adaptive Behavior Composite score, and eye tracking movements in patients with ASD^78^. Therefore, *Lr* in its biofilm state may have positive cognitive effects in humans.

Taken together, our current pig study, our previous rodent studies, and the recent SB-121 ASD trial in adults provide a solid ground for a clinical trial in neonates at risk of developing NEC.

## Methods

### Bovine colostrum

Bovine colostrum was obtained fresh frozen from Jersey cows at the Ohio State University (OSU) Waterman Farm (Columbus, OH) and stored at − 20 °C. Colostrum quality was assessed by measuring immunoglobulin G (IgG) levels using the bovine IgG enzyme-linked immunosorbent assay (ELISA) kit (Bethyl Laboratories Inc., Montgomery, TX). Briefly, samples of frozen colostrum from each batch were thawed and centrifuged three times at 10,000×*g* for 20 min at 4 °C, with the supernatant collected from each step. Supernatants were diluted to 1:500,000 and 1:1,000,000 for ELISA. ELISA was performed in duplicate in 96-well plates using strepavidin-conjugated horseradish peroxidase and chromogenic 3,3′,5,5′-tetramethylbenzidine substrate. Absorbance was measured at 450 nm using the Spectramax M2 microplate and SoftMax Pro 5.4 software (Molecular Devices, San Jose, CA). IgG levels were calculated using a standard curve generated by the SoftMax Pro 5.4 software.

### Lr preparation

Scioto Biosciences, Inc. (Indianapolis, IN) in partnership with List Labs (San Jose, CA) supplied lyophilized *Lr Kandler* under previously reported conditions^78^. Lyophilized *Lr Kandler* was resuspended in 10 mL de Man, Rogosa, and Sharpe (MRS) broth (Fisher Scientific, Pittsburgh, PA) and centrifuged at 3220×*g* for 10 min. The *Lr* pellet was then washed twice in an equal volume of sterile 0.9% saline and isolated again by centrifugation. Final resuspension of the pellet was performed using sterile 0.9% saline to achieve a target concentration of 1.85 × 10^9^ CFU/mL per animal. This resuspension of *Lr* is referred to as *Lr* in its planktonic state.

### Preparation of *Lr* in its biofilm state

*Lr* in its biofilm state was prepared by incubating planktonic *Lr* with anhydrous, biocompatible dextranomer microspheres (DM) (Sephadex G-25 Superfine, Cytiva, Marlborough, MA). DMs were hydrated overnight with a filter sterilized 1 M maltose solution at 4 μl/mg at RT to produce DM-maltose. *Lr* was incubated with DM-maltose at RT for 30 min, adding 1.85 × 10^9^ CFUs of *Lr*/mL of 0.9% sterile saline to 18.5 mg of DM, to induce biofilm production upon adherence of *Lr* to the surface of DM.

### Pig NEC model and administration of *Lr*

The enteral feed-only model of NEC was used as we have previously described^28^. All animal studies were carried out in accordance with and approved by the Research Institute at Nationwide Children’s Hospital Institutional Animal Care and Use Committee (IACUC) (protocol #AR18-00062). All housing and feeding guidelines followed the Guide for the Care and Use of Lab Animals (8th edition). All experiments with our animals were performed in accordance with relevant guidelines and regulations. Reporting in the manuscript follows the recommendations in the ARRIVE guidelines. White Yorkshire x Landrace sows were acquired from Oak Hill Genetics (Ewing, IL) and acclimatized for 5 days prior to terminal Cesarean section (C-section). C-sections were performed on gestational day 104 (full term gestation being 114 days), equivalent to approximately 32 weeks gestation in premature human infants^33^.

Prior to C-section, anesthesia was induced in sows using an intramuscular (IM) cocktail of Telazol (tolazoline/zolazepam, 0.4–1 mg/kg), ketamine (1–2.5 mg/kg), and xylazine (1–2.5 mg/kg), followed by a dose of IM glycopyrrolate (0.01 mg/kg). Sows were intubated with an appropriately sized endotracheal tube (average 9–11 endotracheal tube). Anesthesisa was maintained using 1–4% isoflurane gas inhalant. The abdomen was prepped and draped, and an infraumbilical laportomy incision was made. The uterus was exteriorized and each piglet delivered individually with the umbilical cord milked towards the piglet before the cord was clamped and ligated. Once the C-section was complete, the sow was euthanized with an intravenous (IV) overdose of Euthasol^®^ (1 mL/4.5 kg, Virbac AH, Inc., Fort Worth, TX) and a thoracotomy was made as a secondary method of euthanasia per the American Veterniary Medical Association.

After delivery, piglets were physically stimulated, and their nares and mouths were cleared of amniotic fluid using bulb suction. When the piglets began breathing spontaneously, they received 1 mL of IM iron dextran (Vedco, Saint Joseph, MO), 1 drop of sublingual Doxapram (WEST-WARD, Eatontown, NJ), and sublingual glucose paste (Insta-Glucose, Valeant Pharmaceuticals, Bridgewater, NJ). Piglets received suppemental oxygen at 100% using a non-rebreathing reservoir mask. Once piglets were breathing independently, they were placed into small animal intensive care units (Suburban Surgical Co, Wheeling, IL) set to approximately 37.8 °C and FiO2 at 40%.

After resuscitation, piglets were weighed and sexed. A 6-French transbuccal orogastric feeding tube (Cardinal Health, Dublin, OH) was inserted and secured with 3–0 polypropylene suture at the 18–20 cm mark to the cheek. After confirmation of correct placement of the tube by gastric aspiration or auscultation, piglets received 10 mL of dextrose (25 g) resuspended in 500 mL of Pedialyte (Abbott, Columbus, OH). Goal feeds for all piglets consisted of 120–150 mL/kg/day of bovine colostrum or Neocate Jr (Nutricia, Zoetermeer, Netherlands), prepared per manufacturer’s instructions, every 3 h. Feeds were increased in a scaled fashion over the first two days, with feeds given at 60% goal volume on day 1 and 75% goal volume on day 2. On both days, the feeds were supplemented with Pedialyte to reach 100% goal volume. By day 3, all piglets were receiving 100% of goal volume. Piglets in the Colostrum group received bovine colostrum diluted to 50% with sterile water for the entire experiment, since we previously found that higher concentratons of bovine colostrum led to gastric bezoar formation^28^. Piglets in the NEC + Saline, NEC + *Lr* (biofilm), and NEC + *Lr* (planktonic) groups initially received 12–24 h of 50% colostrum diluted with sterile water followed by administration of Neocate Jr formula to exacerbate induction of NEC. We excluded animals from data collection if they required euthanasia within the first 24 h of life which was established a priori*,* since these deaths likely occurred too soon for NEC to have developed, and were generally due to pulmonary or other causes.

Piglets were randomized into experimental goups by manually sorting them into different groups using Microsoft Excel (Redmond, WA), while maintaining equal weight distribution. Piglets were randomized regardless of sex. Piglets in the NEC + *Lr* (biofilm) or NEC + *Lr* (planktonic) group received 1 mL of *Lr*-DM-maltose or planktonic *Lr* daily respectively, whereas piglets in the NEC + Saline cohort received 1 ml of sterile saline daily, throughout the 5 days of experimentation. These treatments were initially administered within 4–6 h of birth, and then between feeds throughout the study. All investigators responsible for assessing experimental endpoints (CSS, gross injury scores, histologic injury scores, and bacterial translocation) were blinded to the randomization process, daily probiotic/saline administrations during the experiment, and outcome assessment. Once all experimental endpoints were finalized, then all investigators were unblinded for data analysis.

### Clinical sickness scores (CSS)

Piglets were assigned a CSS from 0 to 8 every 3 h during feeds. CSS is a summation of four different components: motor/tone, verbal, alertness, and body color^28^. Each criterion was graded from 0 to 2 depending on severity as follows: Motor/tone: 0 = ambulation and good tone, 1 = recumbent and good tone, 2 = recumbent and poor tone; Verbal: 0 = vocal, 1 = vocal with stimulation, 2 = non-vocal; Alertness: 0 = awake and alert, 1 = responsive to stimuli, 2 = unresponsive to stimuli; Body color: 0 = pink, 1 = pale, 2 = gray. Piglets had a positive CSS if they had a score ≥ 5 out of a maximum of 8 within the last 12 h of life. If a piglet was euthanized prior to the end of the experiment, then it received a score of 8 for the remainder of the experiment.

### Euthanasia and necropsy

Piglets met immediate humane euthanasia criteria for weight loss > 25%, a single temperature reading > 40.5 °C, or extreme lethargy without improvement with resuscitation. Piglets also met humane euthanasia criteria if two of the following were present over two consecutive feeds: (1) > 20% weight loss, (2) temperature > 40 °C, (3) significant lethargy/decreased activity without improvement following resuscitation, (4) bloody diarrhea, (5) abdominal distension, (6) emesis, or (7) rapid, labored, or shallow breathing. Euthanasia criteria were monitored every 30–60 min depending on the health status of the piglets. Once humane endpoint criteria for euthanasia were met, or at the end of the 5-day experiment, piglets were euthanized with IM ketamine (0.3–0.6 mL/kg, Hikma, Berkeley Heights, NJ) and xylazine (0.1–0.2 mL/kg, Akorn, Lake Forest, IL), followed by intraperitoneal (IP) Euthasol^®^ (1–2 mL/kg, Virbac AH, Inc., Fort Worth, TX). Piglets were then prepared for necropsy in a sterile fashion. BD ChloraPrep™ (Becton, Dickinson and Company, Franklin Lakes, NJ) was applied to the chest and abdomen, and thoracotomy and laparotomy were performed. Samples of liver, spleen, and mesenteric lymph nodes were collected in a sterile fashion for bacterial translocation. The lungs and intestines were examined, and the intestines were graded according to the gross injury scoring system that we adapted from the literature^28,81^. A score of 1 was given for no injury, 2 for hyperemia in a small section of intestine, 3 for hyperemia and edema throughout the intestine or hemorrhage in a small section of intestine, 4 for hemorrhage throughout the intestine, 5 for necrosis in a small section of intestine or pneumatosis intestinalis, and 6 for transmural necrosis, pneumatosis intestinalis throughout the intestine, or intestinal perforation^28,33,81^. A gross injury score of ≥ 4 was considered to be consistent with NEC^28^. Intestinal samples from the proximal, mid, and distal small bowel and colon were collected for histologic examination. In addition, colonic contents were obtained for 16S rRNA sequencing analysis. Finally, a craniotomy was performed, and the brain was harvested and dissected to collect the prefrontal cortex (PFC) for immunohistochemistry (IHC).

### Intestinal histologic injury grading

Intestinal tissues were fixed for at least 24 h in 10% formalin (Fisher Scientific, Pittsburgh, PA), followed by incubation in 70% ethanol (Fisher Scientific, Waltham, MA). Intestinal samples were paraffin-embedded, sectioned at 5 μm thickness, and stained with hematoxylin and eosin (H&E). Slides were reviewed by at least two independent and blinded reviewers, including a board-certified pediatric pathologist. Using a previously published histologic injury grading system, a score from 1 to 5 was given to the duodenum, jejunum, ileum, and colon, with grades 1–2 representing no histologic injury, grades 3–4 representing moderate histologic injury, and grade 5 representing severe injury^28,82^. With respect to the small intestine, a score of 1 represents no damage, 2 represents damage to epithelial cells at the tips of villi with the majority of villi intact, 3 represents necrosis of epithelial cells to the mid-villus level, 4 represents necrosis of entire villi, occasional villi, or pneumatosis, and 5 represents transmural necrosis, scant villi, or widespread pneumatosis^28^. Regarding the colon, a score of 1 is consistent with no damage, 2 represents minimal mucosal breakage, 3 represents mucosal sloughing with erythrocyte infiltration, 4 represents pneumatosis with incomplete mucosal necrosis or complete mucosal necrosis without pneumatosis, and 5 represents pneumatosis with transmural necrosis^28^. Images were obtained using Olympus U-TV0.5XC-3 (Tokyo, Japan). The grade seen in the majority of a given segment of intestine was assigned for that specific section of intestine. The most severe histologic injury from any intestinal segment (proximal, mid, distal small bowel, or colon) was assigned as the final histologic score for that piglet.

### Brain immunohistochemistry

After brains were harvested, the PFC regions were fixed in 10% neutral buffered formalin for 24 h at RT. Paraffin-embedded tissues were sectioned at 5 μm thickness, deparaffinized in xylene, and rehydrated in ethanol. Antigen retrieval was performed by incubating slides in 10 mM sodium citrate buffer (pH 6.0) within a boiling water bath for 40 min. Endogenous peroxidase was quenched by incubating with 0.3% hydrogen peroxide in methanol for 30 min. Tissue sections were blocked in buffer containing 1% bovine serum albumin (BSA), 2% goat serum, 0.1% Triton, and 0.05% Tween 20 for 2 h at RT. The primary antibodies used were anti-ionized calcium binding adaptor molecule 1 (Iba-1) (#019-19741, FujiFilm Wako, Richmond, VA) and myelin basic protein (MBP) (#78896, Cell Signaling, Danvers, MA), both at 1:500 dilution. Samples were incubated with primary antibody at 4 °C overnight. The following day, samples were incubated with 1:500 biotinylated anti-rabbit IgG secondary antibody (Jackson ImmunoResearch, West Grove, PA) at RT. Samples were then treated with Avidin–Biotin Complex (ABC) horseradish peroxidase (HRP) reagents (Vector Lab, Newark, CA) for 30 min at RT and visualized by incubation with 3,3’-Diaminobenzidine tetrahydrochloride (DAB) (Sigma, Burlington, MA), followed by mounting with DPX solution (Sigma, Burlington, MA). For negative controls, samples were incubated with secondary antibody only with no primary antibody added.

For Iba-1 quantification, a minimum of 4 random images at 40× magnification were taken using a Zeiss Axio A1 microscope equipped with Axiovision software (Carl Zeiss Microscopy GmbH, Jena, Germany). Activated microglia from each image were counted manually in a blinded fashion by two independent reviewers. For MBP quantification, a minimum of 3 random images were obtained with the ECLIPSE Ti2 inverted microscope at 40× magnification equipped with the NIS-Elements workstation (Nikon, Tokyo, Japan). The percent surface area stained was quantified in a blinded fashion using ImageJ software (National Institutes of Health, Bethesda, MD).

### Bacterial translocation

Samples of liver, spleen, and mesenteric lymph nodes were collected upon necropsy, snap frozen, and stored at − 80 °C until use. Once ready for assay, samples were thawed on ice, individually weighed, mechanically homogenized, and then plated on Brain Heart Infusion (BHI) plates overnight at 37 °C. The following day, colonies were counted and reported as CFU per mg of tissue.

### Definitive NEC (D-NEC) score

As we described previously^28^, at least 3 of the following 4 criteria were required for a piglet to be diagnosed with D-NEC: CSS of ≥ 5 out of 8 in the last 12 h of life, gross injury score of ≥ 4 out of 6, histologic injury score of ≥ 3 out of 5, and bacterial translocation to ≥ 2 internal organs (liver, spleen, and mesenteric lymph nodes with ≥ 1 CFU/mg of tissue). Historically, gross and/or histologic injury were used by others to determine whether a premature piglet had NEC^32^. In the development of our model, we found that gross or histologic injury alone did not correlate well with the clinical status of the piglet. Therefore, we developed a novel multifactorial D-NEC scoring system similar to Bell’s criteria that is used to diagnose NEC in premature neonates to better define the disease in our model^83^. CSS reflects the clinical status of the piglet, and bacterial translocation serves as a surrogate for intestinal epithelial integrity. Death associated with D-NEC throughout the experiment was determined by including only piglets that were euthanized during the experiment and had a positive D-NEC score.

### DNA extraction and 16S ribosomal (rRNA) gene sequencing

Colonic contents were aseptically collected from piglets at sacrifice and stored at − 80 °C until use. Approximately 100 mg of colonic contents were used for DNA extraction with the QIAamp DNA Mini Kit (Qiagen, Hilden, Germany) per manufacturer’s instructions with slight modifications. Contents were incubated for 45 min at 37 °C in lysozyme-mutanolysin buffer (pH 8.0) containing 22 mg/mL lysozyme, 0.1 U/mL mutanolys, 20 mM TrisHCL, 1.2% Triton-x (Sigma Aldrich, St. Louis, MO), and 2 mM EDTA (Fisher Scientific, Waltham, MA), followed by homogenization for 150 s with 0.1 mm zirconia beads. Samples were incubated at 95 °C for 5 min with InhibitEx Buffer, then incubated at 70 °C for 10 min with Proteinase K and Buffer AL (Qiagen). Following this step, the QIAamp DNA Mini Kit isolation protocol was followed beginning with the ethanol step. DNA was quantified on the Qubit 2.0 Fluorometer (Life Technologies, Carlsbad, CA) with the dsDNA Broad Range Assay Kit. Total *L. reuteri* and total bacteria were quantified in each sample via qPCR using strain-specific and universal 16S primers, respectively. Primers and qPCR conditions are detailed in Supplementary Table S1 online. Samples were stored at − 20 °C prior to 16S rRNA gene sequencing.

DNA from representative piglets from Experiment 1 was submitted to the Genomic Services Core at the Institute for Genomic Medicine at Nationwide Children’s Hospital (Columbus, OH) for library preparation and high-throughput sequencing. Paired-end (300 nt forward and reverse) sequences of the V4 hypervariable region of the 16S rRNA gene (515F-806R) were generated by Illumina MiSeq. Quantitative Insights Into Microbial Ecology (QIIME) 2.0^84^ and DADA2^85^ were utilized for downstream amplicon processing, quality control, diversity analyses, and taxonomic assignment with a trained classifier built from the SILVAv138.99 ribosomal RNA database^86,87^. For quality control, sequences were truncated from the 3′ end to 180 nt (forward reads) or 130 nt (reverse reads) to achieve an average quality score of at least 20, and the first 20 nt or 25 nt were trimmed from the 5′ end of forward and reverse reads, respectively. Sequences that did not meet these criteria were discarded. After quality control, taxa denoted as eukaryotic, “unassigned”, “chloroplast”, “mitochondria”, or not annotated beyond the phylum level were filtered from the dataset. Compositional alpha and beta diversity were assessed at a sequencing depth of 8,900 sequences per sample using the core-metrics-phylogenetic QIIME 2.0 pipeline, sequences with fewer reads were omitted from diversity analyses. Evenness, richness (number of observed species), Shannon Diversity^88^, Simpson Diversity^89^, and Faith’s Phylogenetic Diversity (Faith PD)^90^ were calculated to measure alpha diversity within samples based on treatment. The Jaccard^91^, UniFrac^92^, and Bray-Curtis^93^ distance matrices were used to assess beta diversity, and the EMPeror software package^94^ was used to construct three-dimensional principal coordinate analysis (PCoA) plots to visualize differences based on treatment and occurrence of D-NEC. To quantify changes in differential abundance between treatments, data were filtered to retain features that were present at a minimum of 0.01% abundance in at least 10% of samples. Family level relative abundances were collapsed across treatment for representative bar plots using the q2-taxa plugin.

### Statistical analyses

Differences in percent gross injury, histologic injury, bacterial translocation, D-NEC scores, activated microglial counts, and MBP-stained surface area were evaluated using the Kruskal–Wallis test. CSS at different timepoints was evaluated using 2-way analysis of variance (ANOVA). Percent death associated with D-NEC was analyzed using the log-rank (Mantel-Cox) test with Bonferroni correction. All statistical analyses were performed using GraphPad Prism 9.0.0 (GraphPad Software, Boston, MA). *p* < 0.05 was considered statistically significant in all other analyses.

Comparisons for evenness, richness, and Faith PD alpha diversity metrics were made in QIIME2.0 with the Kruskal–Wallis test. Differences in beta diversity Jaccard distances were analyzed in QIIME2.0 by permutational multivariate analysis of variance (PERMANOVA) with 999 randomizations of the data. Differences in differential abundance between treatments at the various taxonomic levels were assessed using ALDEx2 package in R with Wilcoxon *p* values adjusted via the Benjamini–Hochberg method to control false discovery rate (FDR; *p*.adj < 0.05)^95–97^. *p* < 0.05 was considered statistically significant in all analyses.

### Supplementary Information

Supplementary Figures.

Supplementary Information.

Supplementary Tables.

## Data Availability

The datasets generated during and/or analyzed during the current study are available in the Sequence Read Archive (SRA) with the following BioProject accession number: PRJNA975191. https://urldefense.com/v3/, http://www.ncbi.nlm.nih.gov/bioproject/975191__;!!NiUAmZJ8c1GNWg!Ul28hYsvFAafKIarrCvCSCg4Rut9wzAtW0zSiT6f6rL3f_SmcTBI75WjS47D-Rd71OPy9ykBcovjd27RJ8FWssARI-JsJx6DHMe7rOD0L7QQvQ$.
