# Supplementary material for: Superior performance of biofilm versus planktonic Limosilactobacillus reuteri in protection of the intestines and brain in a piglet model of necrotizing enterocolitis
Source: Sci Rep. 2023 Oct 23;13:17740. doi: 10.1038/s41598-023-44676-5 (PMC10593788; doi:10.1038/s41598-023-44676-5)
Supplement: Supplementary file 1 — Supplementary Figures. [file 41598_2023_44676_MOESM1_ESM.pdf]

Superior Performance of Biofilm *vs.* Planktonic *Limosilactobacillus reuteri* in Protection of the Intestines and Brain in a Piglet Model of Necrotizing Enterocolitis

Samantha J. Wala, Nitin Sajankila, Mecklin V. Ragan, Audrey F. Duff, Joseph Wickham, Samuel G. Volpe, Yijie Wang, Miriam Conces, Zachary Dumbauld, Nanditha Purayil, Siddharth Narayanan, Adrian Rajab, Belgacem Mihi , Michael T. Bailey, Steven D. Goodman, Gail E. Besner

Supplementary Figure S1

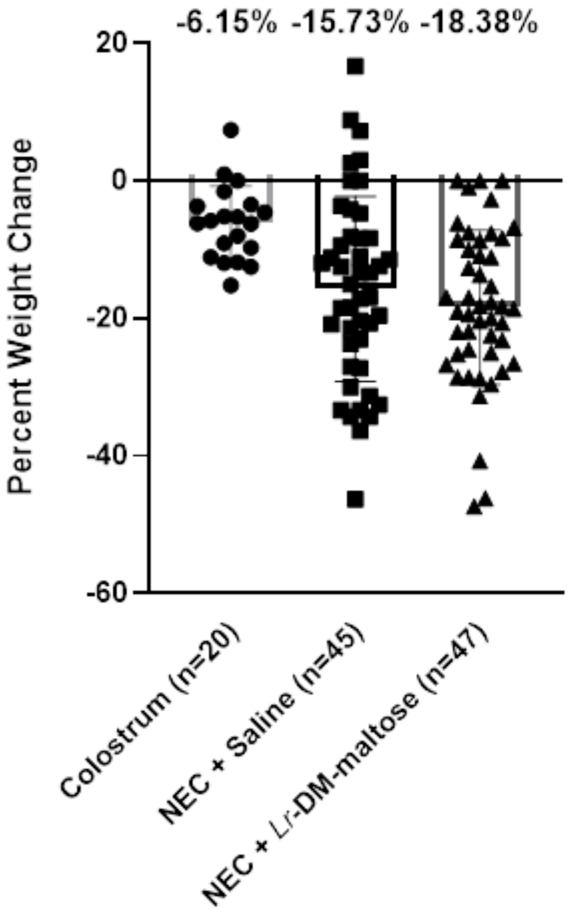

Supplementary Figure S1. Average percent weight change in Experiment 1. The average differences in weight between birth and at time time of euthanasia in the Colostrum, NEC + Saline, and NEC + *Lr*-DM-maltose groups are shown. Points indicate quantification for each individual animal, columns indicate the mean, and error bars represent standard error of the mean (SEM).

Supplementary Figure S2

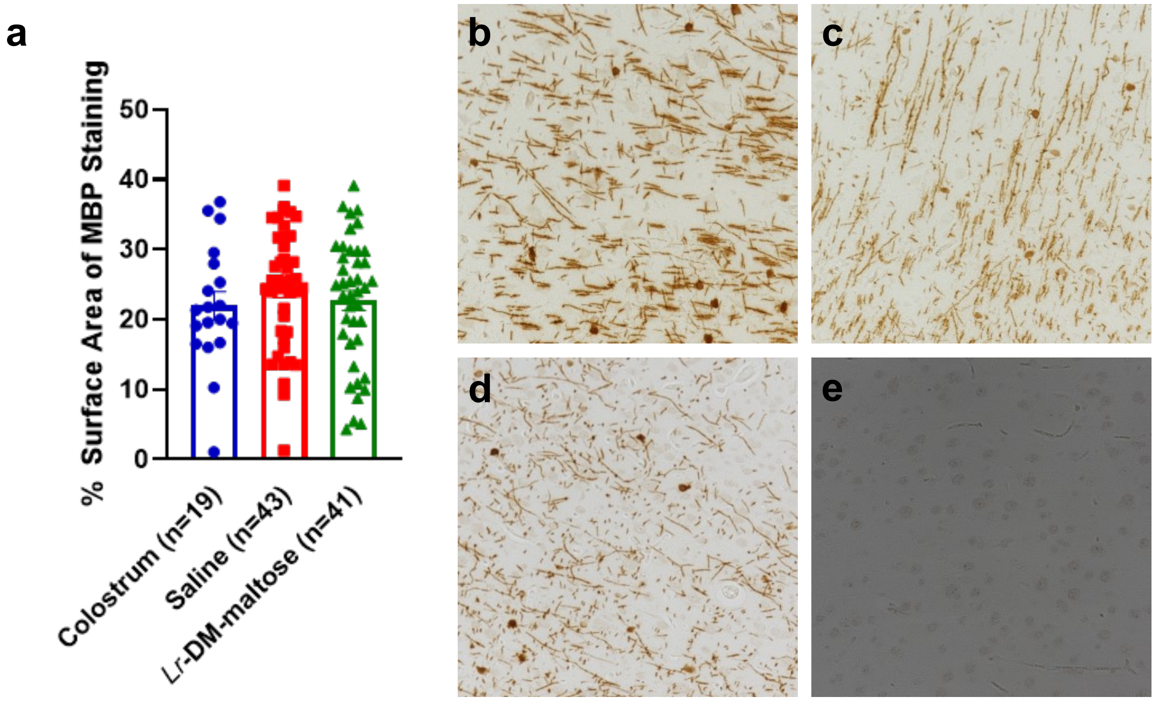

Supplementary Figure S2. Myelin basic protein (MBP) expression in prefrontal cortex (PFC) from Experiment 1. (**a**) Mean percent surface area stained for MBP in PFC. Points indicate quantification for each individual animal, columns indicate the mean, and error bars represent standard error of the mean (SEM). The Kruskal-Wallis test was used. Images of MBP stained PFC at 40X magnification in the (**b**) Colostrum, (**c**) NEC + Saline, and (**d**) NEC + *Lr*-DM-maltose groups. (**e**) represents the negative control.

Supplementary Figure S3

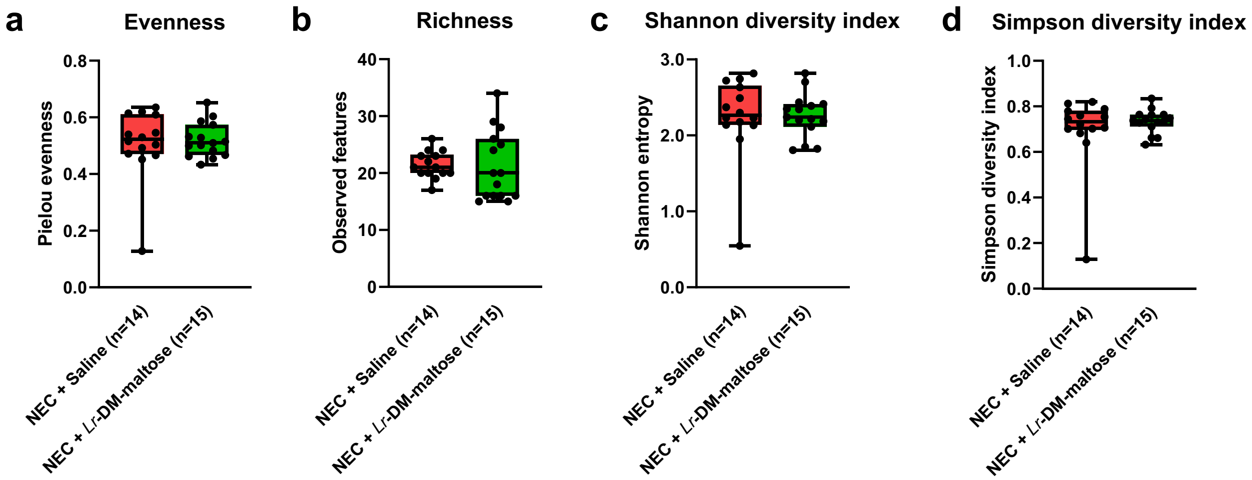

Supplementary Figure S3. Additional alpha diversity metrics. (**a**) Evenness (p=0.600), (**b**) richness (number of observed features; p=0.496), (**c**) Shannon diversity index (p=0.662), and (**d**) Simpson diversity index (p=0.827) within treatment. Pairwise comparisons between groups were generated in QIIME 2.0 with the Kruskal-Wallis (p<0.05). Data are represented as box and whisker plots that denote minimum, maximum, and interquartile range values.

Supplementary Figure S4

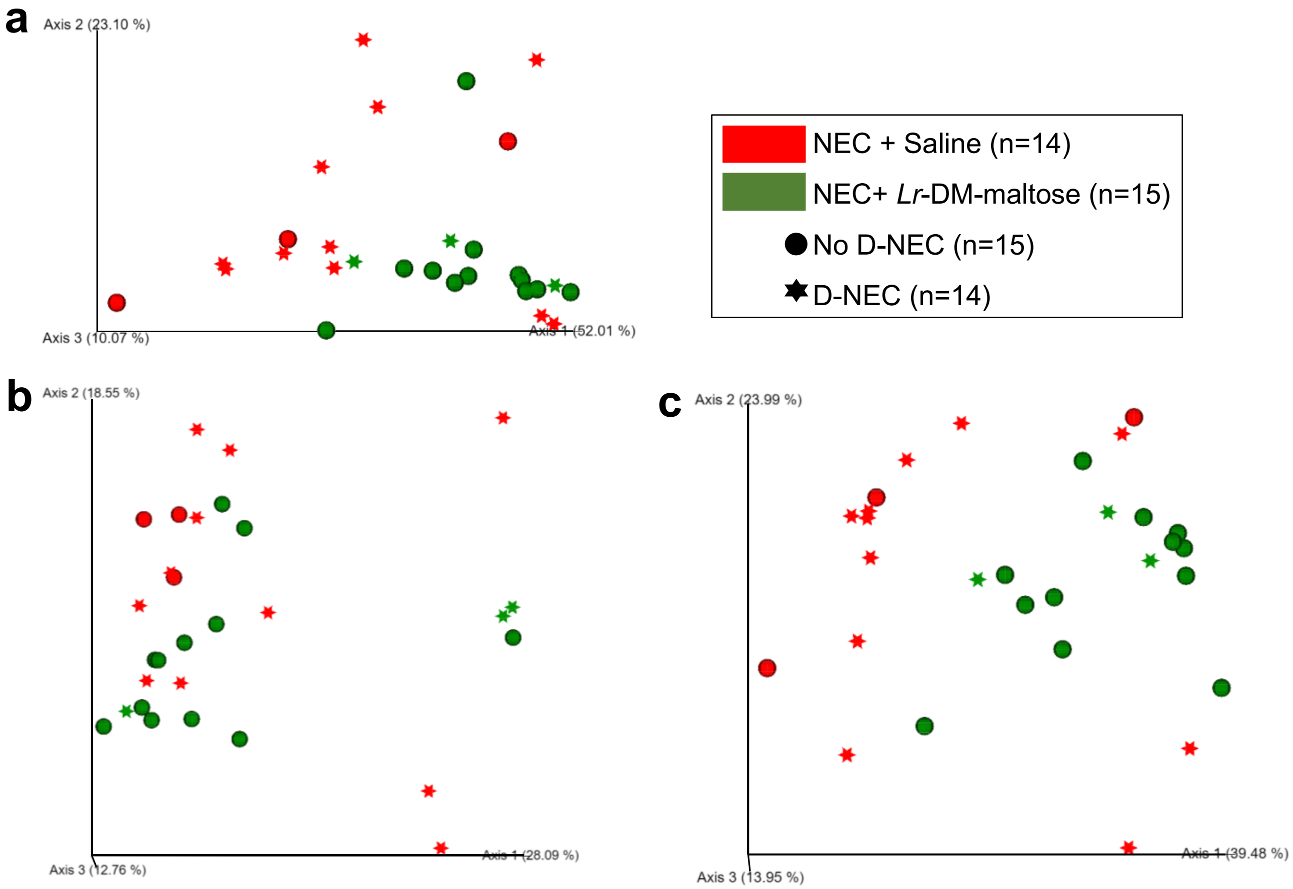

Supplementary Figure S4. Additional beta diversity indices. Principal coordinate analysis (PCoA) plots of beta diversity clustering. (**a**) Weighted UniFrac distances based on treatment (p=0.007) and occurrence of Definitive NEC (D-NEC) (p=0.284). (**b**) Unweighted UniFrac distances for effects of treatment (p=0.020) and occurrence of D-NEC (p=0.102). (**c**) Bray-Curtis distances for effects of treatment (p=0.003) and occurrence of D-NEC (p=0.109). All distance matrices were analyzed in QIIME 2.0 by PERMANOVA using 999 randomizations of the data.

Supplementary Figure S5

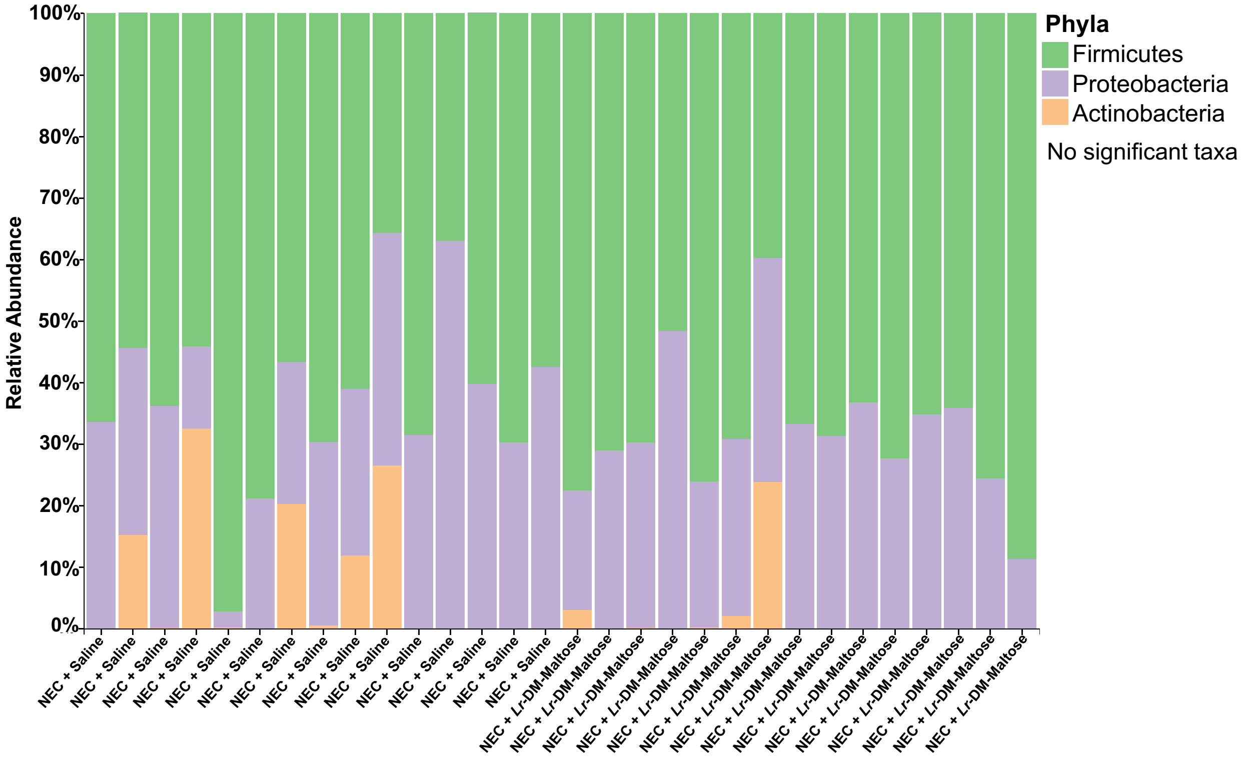

Supplementary Figure S5. Phylum Level Differential Abundance Within Individual Samples. Phylum level taxa distribution within treatment. Bars represent mean relative percentage of each corresponding bacterial phyla. No significant differences in differential abundance were determined via ALDEX2 in R, Wilcoxon *p*-values were Benjamini-Hochberg adjusted (FDR significance level = 0.05).

Supplementary Figure S6

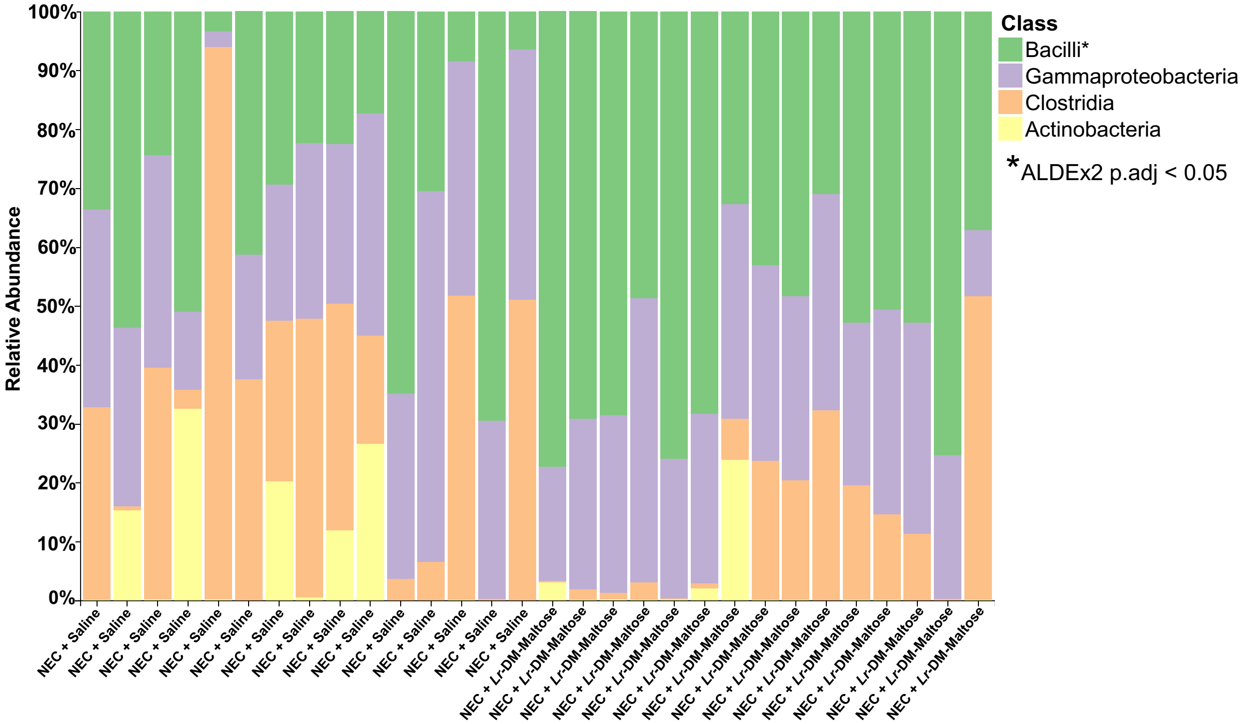

Supplementary Figure S6. Class Level Differential Abundance Within Individual Samples. Class level taxa distribution within treatment. Bars represent mean relative percentage of each corresponding bacterial class. Significant differences in differential abundance were determined via ALDEX2 in R, Wilcoxon p-values were Benjamini-Hochberg adjusted (FDR significance level = 0.05).

Supplementary Figure S7

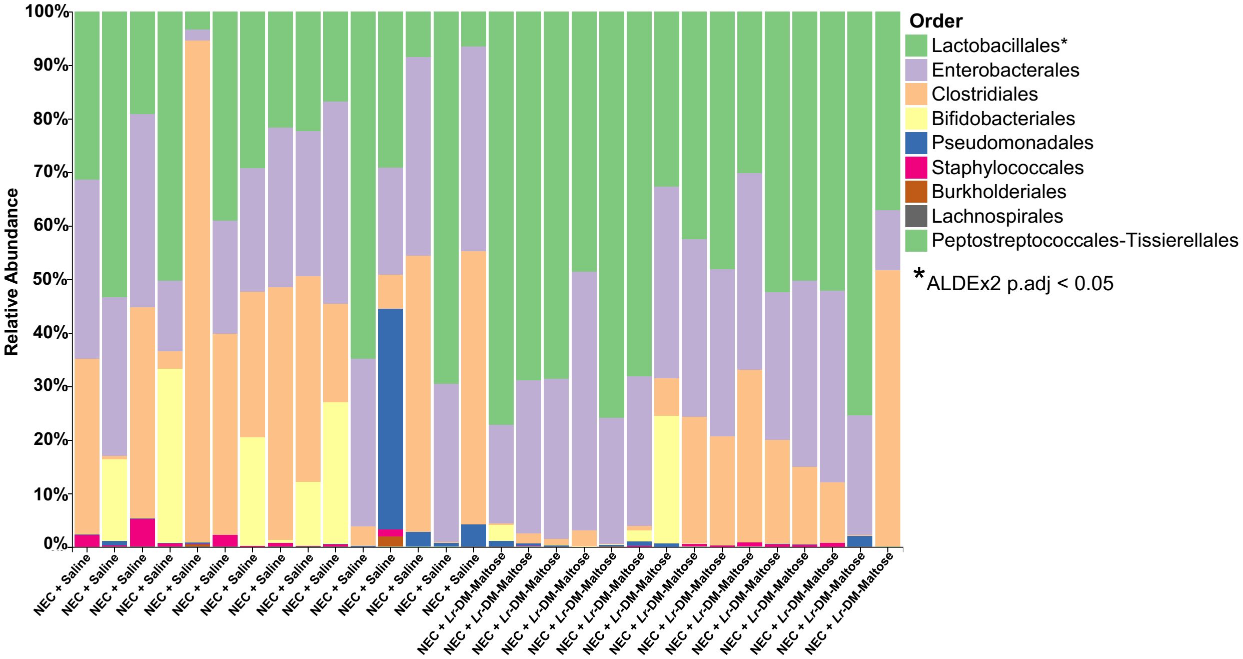

Supplementary Figure S7. Order Level Differential Abundance Within Individual Samples. Order level taxa distribution within treatment. Bars represent mean relative percentage of each corresponding bacterial order. Significant differences in differential abundance were determined via ALDEX2 in R, Wilcoxon *p*-values were Benjamini-Hochberg adjusted (FDR significance level = 0.05).

Supplementary Figure S8

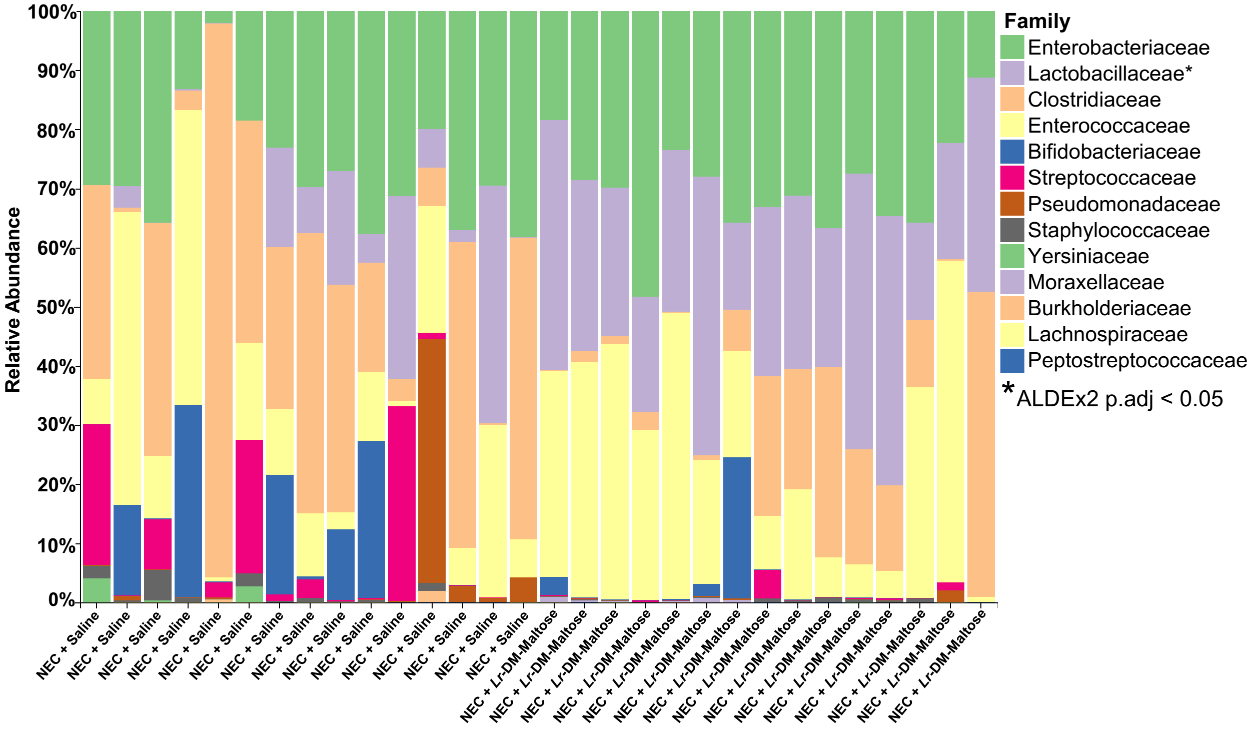

Supplementary Figure S8. Family Level Differential Abundance Within Individual Samples. Family level taxa distribution within treatment. Bars represent mean relative percentage of each corresponding bacterial family. Significant differences in differential abundance were determined via ALDEX2 in R, Wilcoxon *p*-values were Benjamini-Hochberg adjusted (FDR significance level = 0.05).

Supplementary Figure S9

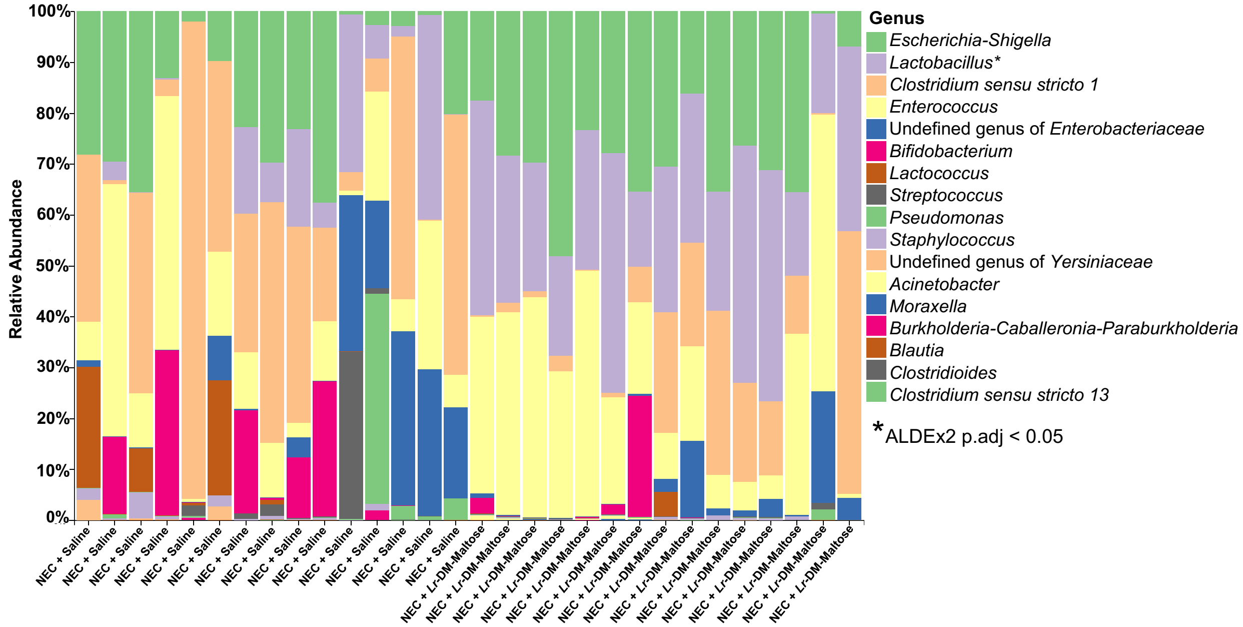

Supplementary Figure S9. Genus Level Differential Abundance Within Individual Samples. Genus level taxa distribution within treatment. Bars represent mean relative percentage of each corresponding bacterial genus. Significant differences in differential abundance were determined via ALDEX2 in R, Wilcoxon *p*-values were Benjamini-Hochberg adjusted (FDR significance level = 0.05).

Supplementary Figure S10

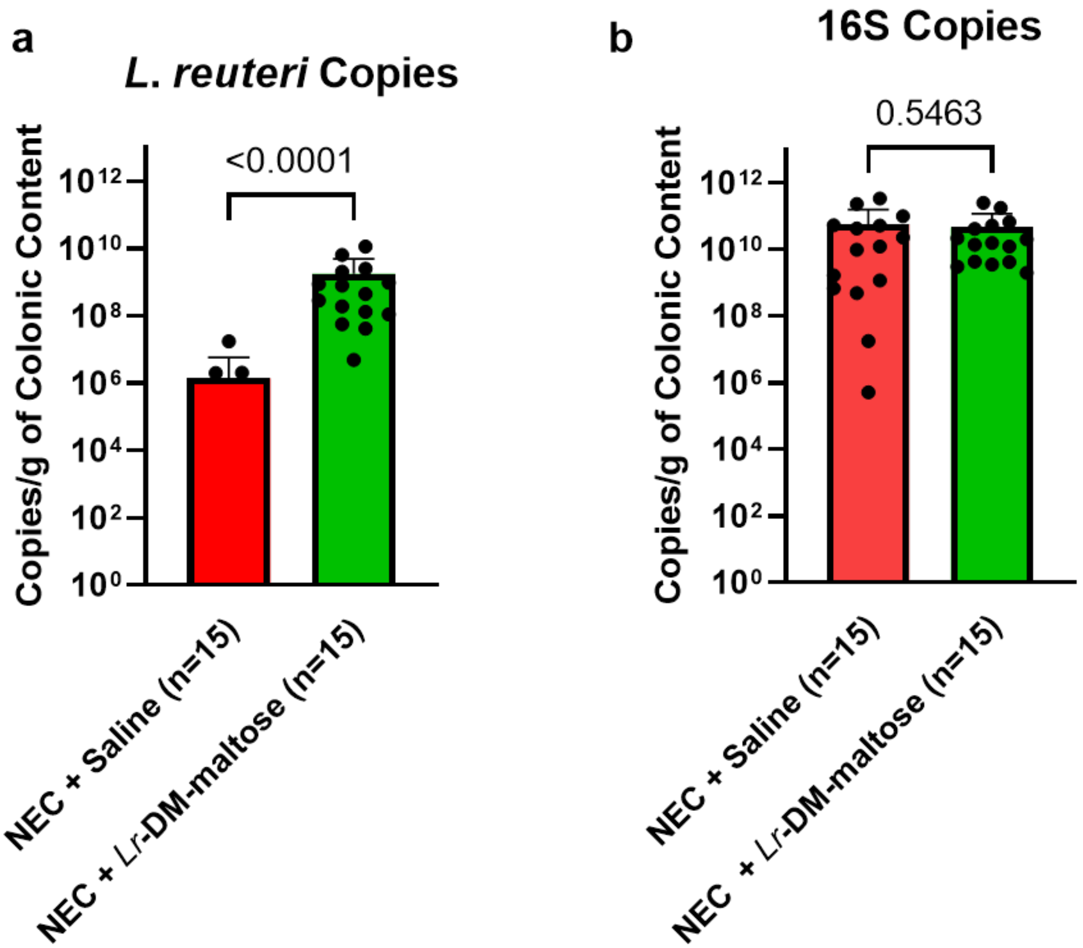

Supplementary Figure S10. Absolute bacterial quantification. (**a**) Total *L. reuteri* or (**b**) total bacteria copies per gram of colonic content. Quantification of *L. reuteri* and total bacteria was performed via qPCR with strain-specific or universal 16S primers, respectively, in the NEC + Saline and NEC + *Lr*-DM-maltose groups. Columns indicate the mean, and error bars represent standard deviation. Values on the x-axis are represented on a logarithmic scale and therefore values of zero are not shown. Differences between treatments were assessed using the Mann-Whitney t-test (p<0.05).

Supplementary Figure S11

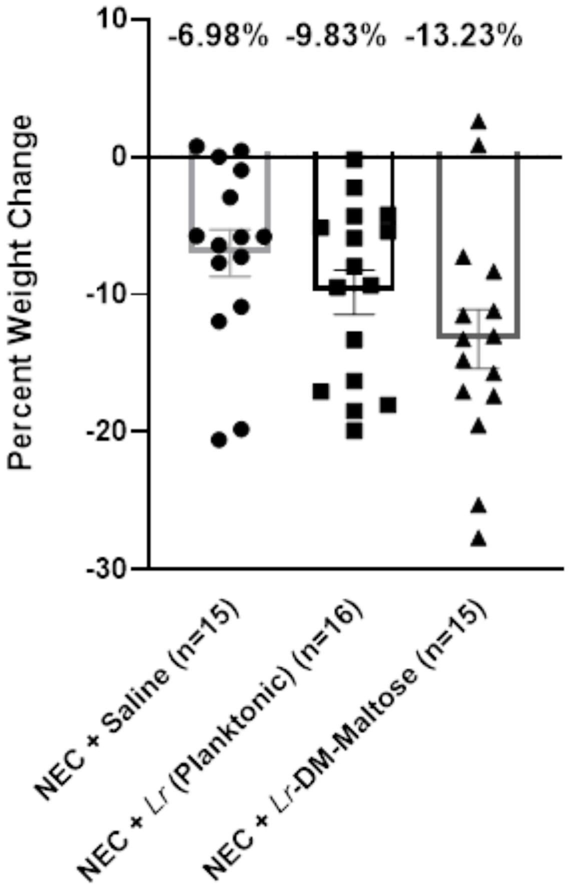

Supplementary Figure S11. Average percent weight change in Experiment 2. The average differences in weight between birth and at time time of euthanasia in the NEC + Saline, NEC + *Lr* (Planktonic), and NEC + *Lr*-DM-maltose groups are shown. Points indicate quantification for each individual animal, columns indicate the mean, and error bars represent standard error of the mean (SEM).
