# Supplementary material for: Superior performance of biofilm versus planktonic Limosilactobacillus reuteri in protection of the intestines and brain in a piglet model of necrotizing enterocolitis
Source: Sci Rep. 2023 Oct 23;13:17740. doi: 10.1038/s41598-023-44676-5 (PMC10593788; doi:10.1038/s41598-023-44676-5)
Supplement: Supplementary file 3 — Supplementary Tables. [file 41598_2023_44676_MOESM3_ESM.pdf]

Superior Performance of Biofilm *vs.* Planktonic *Limosilactobacillus reuteri* in Protection of the Intestines and Brain in a Piglet Model of Necrotizing Enterocolitis

Samantha J. Wala, Nitin Sajankila, Mecklin V. Ragan, Audrey F. Duff, Joseph Wickham, Samuel G. Volpe, Yijie Wang, Miriam Conces, Zachary Dumbauld, Nanditha Purayil, Siddharth Narayanan, Adrian Rajab, Belgacem Mihi , Michael T. Bailey, Steven D. Goodman, Gail E. Besner

Supplementary Table S1

Primer	Sequence (5'-3')	Final Concentration
L. reuteri_F	GGAATATCAGGCTCACGGAT	200nM
L. reuteri_R	ACTGGCTTATGGCGTTTTTC	200nM
16S_F	CGGTGAATACGTTCYCGG	1.5µM
16S_R	GGWTACCTTGTTACGACTT	1µM

L. reuteri Primer Conditions

	Temperature	mm:ss
	50°C	2:00
	95°C	2:00
45x	95°C	0:15
	53.8°C	0:15
	72°C	0:15
	Melt Curve	

16S Primer Conditions

	Temperature	mm:ss
	50°C	2:00
	95°C	10:00
40x	95°C	0:15
	56°C	1:15
	Melt Curve	

Supplementary Table S1. *L. reuteri* and universal 16S bacterial qPCR primers and conditions. All reactions were carried out using SYBR green chemistry in 20µL reaction volumes (18µL master mix and 2µL of DNA template (5ng/µL)).
